# Magnetite Concentration-Driven Evolution of Microstructure and Property Tuning in Alginate Hydrogels

**DOI:** 10.3390/gels12090786

**Published:** 2026-09-01

**Authors:** Vasily I. Mikhaylov, Mikhail A. Torlopov, Ilia S. Martakov, Philipp V. Legki, Kirill S. Vavrinchuk, Pavel A. Markov, Natalia N. Drozd, Kirill A. Cherednichenko, Petr A. Sitnikov

**Affiliations:** 1 Institute of Chemistry of Federal Research Centre “Komi Science Centre of the Ural Branch of Russian Academy of Sciences”, 48 Pervomayskaya St., 167000 Syktyvkar, Russia; tanlan799@gmail.com (M.A.T.); philipp2374372@mail.ru (P.V.L.); kirill.vavrinchuk@inbox.ru (K.S.V.);; 2 National Medical Research Centre for Rehabilitation and Balneology, 32 Novy Arbat, 121099 Moscow, Russia; p.a.markov@mail.ru; 3 National Medical Research Center for Hematology, 4 Novy Zykovsky Passage, 125167 Moscow, Russia; nndrozd@mail.ru; 4 Department of Physical and Colloidal Chemistry, National University of Oil and Gas “Gubkin University”, 65 Leninsky Prospekt, 119991 Moscow, Russia; cherednichenko.k@gubkin.ru

**Keywords:** alginate hydrogels, cell adhesion, fibroblasts, magnetite

## Abstract

In this study, the effect of citrate-modified magnetite nanoparticle (MNP) concentration on the microstructure and properties of composite alginate-containing hydrogels and xerogels was investigated. Data from electron and optical microscopy, FTIR, STA, and mechanical testing demonstrated that MNP concentration directly influences both functional properties and microstructure. Adjusting this parameter allows fine-tuning of the morphology, shifting it from uniform dispersion to particle aggregation, and eventually to sedimentation-driven stratification. At 1 wt%, MNPs are incorporated into the interchain spaces of alginate, leading to partial disruption of the polymer network structure and a reduction in mechanical strength. At 5 wt% MNPs, maximum mechanical strength is achieved due to the formation of oriented clusters uniformly distributed throughout the sample, forming a rigid reinforcing framework. At 10 wt%, the formation of numerous smaller but denser clusters introduces stress concentration sites, leading to a significant decrease in mechanical strength. At 20 wt%, sedimentation of large magnetite clusters during hydrogel formation leads to stratification into a lower MNP-rich reinforcing layer and an upper polymer-rich elastic layer, which remain connected through the continuous alginate matrix, providing a combination of high strength and rigidity with preserved elasticity. All samples showed procoagulant activity, while higher MNP content enhanced fibroblast adhesion and proliferation relative to unmodified alginate. Strong UV–Vis absorption in MNP-containing composites points to their potential to shield bioactive components from unwanted photo-induced degradation. Regulation of structure driven by magnetite concentration opens prospects for the development of alginate matrices with tailored magnetic sensitivity, physicochemical properties, and cytohemocompatibility.

## 1. Introduction

Hydrogels represent an almost ideal intermediary between living tissue and synthetic materials. These flexible three-dimensional polymer structures are distinguished by their hydrophilicity, biocompatibility, biofunctionality, and elasticity [1,2]. One approach to transforming hydrogels from passive implants into active, biointegrable medical platforms involves controlling their texture, physicochemical properties, and cytocompatibility. Nanoparticles are increasingly being applied successfully in this field [3,4,5].

Alginate-based composites have attracted considerable attention from researchers owing to their high chemical stability, biocompatibility, bioresorbability, biodegradability, pH sensitivity, and ease of gelation in the presence of Ca^2+^ cations. However, they are characterised by low mechanical strength and high brittleness [6,7]. Another limitation restricting their use in tissue engineering and regenerative medicine is their extremely poor capacity to support cellular adhesion and proliferation without additional modification [8].

To address these drawbacks, various strategies for modifying alginate hydrogels, including the production of composite materials, are being investigated and applied. One of the most common approaches is the formation of polyelectrolyte complexes with other biopolymers, such as chitosan. This system is proposed for the development of materials suitable for cell growth [9], drug delivery [10], and wound healing applications [11].

The introduction of nanoparticles of various types is an effective method for controlling the mechanical and functional properties of biocomposites, as it enables regulation of the material’s microstructure and strength characteristics [12], increases resistance to degradation in biological environments, and enhances fibroblast infiltration upon contact with the hydrogel [13]. Good dispersion of nanoparticles generally improves strength due to the large surface area available for interaction with the polymer. However, clusters formed by nanoparticles, resulting from their tendency to reduce high surface energy, act as stress concentrators. The presence of clusters comprising strongly bound particles alongside more weakly bound agglomerates is typically regarded as a negative factor, leading to a reduction in the material’s mechanical properties [14]. In previous studies [4], we found that concentration-controlled formation of extensive agglomerates of chitin and cellulose nanocrystals (clusters) results in the development of a secondary percolation network within the alginate matrix. This network not only increases the composite’s rigidity but, more importantly, significantly enhances fibroblast adhesion and proliferative activity by creating an optimal microrelief and chemical environment for the cells. Furthermore, the concentration, particle charge, and nature of active Lewis acid–base sites on the surface play crucial roles in this process. The first factor determines the clustering pathway and the formation of percolation networks. The second influences the mechanism of cluster formation and dictates their size at given particle concentrations. Clustering, in turn, modifies the set of active sites within and on the surface of the aggregates and affects the donor–acceptor properties of the material’s surface. Collectively, these factors not only determine the structural and physicochemical properties of the filled matrices but also govern their cytohemocompatibility.

Magnetite nanoparticles are of considerable interest in the development of composite hydrogels. Currently, there is growing attention towards hydrogels that are sensitive to magnetic fields. The spatial permeability of magnetic fields, compared to other stimuli such as pH and temperature, enables remote and controllable magnetically induced deformation of hydrogel structures containing a magnetic component under the influence of the field. Magnetic components can be categorised into several types, including iron oxides (γ-Fe_2_O_3_ и Fe_3_O_4_), transition metal ferrites (CoFe_2_O_4_, MnFe_2_O_4_, etc.), metals and alloys (Fe, Co, Ni, FePt), and iron nitride particles [15]. Among these, magnetite Fe_3_O_4_ nanoparticles (MNPs) offer advantages such as superparamagnetism, high specific magnetisation values, low toxicity, biocompatibility, stability of properties, a wide range of synthesis methods, environmental safety, and high UV-vis opacity, which provide critical photoprotection for the hydrogel components and underlying biological systems [16]. However, their tendency to agglomerate and susceptibility to oxidation when exposed to humid air or water in their unmodified form have been noted [17]. These drawbacks can be mitigated by surface modification of magnetite particles and/or their incorporation into a polymer matrix. Biopolymers are among the most promising agents for such modification.

Alginate–magnetite composites typically involving alginate-coated nanoparticles or alginate granules with dispersed nanoparticles have been extensively studied for adsorption and photocatalytic applications [18,19,20,21]. Studies exploring the biomedical applications of these systems are comparatively less common and include drug delivery, antibacterial systems, and theranostics. For instance, Fe_3_O_4_@Alg nanoparticles have been used for curcumin delivery [16], while Fe_3_O_4_–Alg hydrogels show antibacterial activity against pathogenic bacteria [22]. Alginate beads loaded with sodium diclofenac and Ag or Fe_3_O_4_ nanoparticles exhibited pH-dependent release and enhanced drug release under an external magnetic field [23]. Similarly, alginate beads with Fe_2_O_3_@SiO_2_ and dextran demonstrated hyperthermic properties and triggered dextran release in an alternating magnetic field, underscoring their theranostic potential [24].

However, studies on the preparation of film materials based on the magnetite–alginate system are scarce, with most research focusing on the mere presence of magnetic nanoparticles (MNPs) rather than the effect of their concentration. For example, alginate films with MNPs (0.5:1 weight ratio) showed magnetically induced release of folic acid [25]. Another study [26] examined alginate films with MNPs in the range of 0–10 wt%. It was reported that the reinforcing effect depends on the presence of plasticizers (glycerol), with significant agglomeration when dried powder was used. However, neither of these studies systematically addressed the broad concentration range or the concentration-driven evolution of microstructure and its correlation with functional properties.

The hypothesis explored in this study is that the functional properties of alginate composites can be controlled through concentration-regulated nanoparticle clustering.

The aim of this study is to comprehensively investigate the influence of the concentration (1–20 wt%) of citrate-modified, never-dried magnetic nanoparticles (MNPs) within the alginate matrix of film hydrogel biomaterials on their microstructure, thermal, spectral, and mechanical properties, as well as their hemocompatibility and cytocompatibility (cytohemocompatibility). This will enable the identification of microstructural changes that determine mechanical performance, and biological characteristics that are important for the rational design of alginate-based biomaterials with tunable properties. Importantly, establishing this fundamental understanding under zero-field conditions is required before applying external magnetic fields to manipulate the composite microstructure.

## 2. Results and Discussion

### 2.1. Alginate and Magnetite Characterization

To produce filled alginate films and hydrogels, a freshly synthesized stable magnetite hydrosol, with particle surfaces modified by citric acid, was employed [27]. The modified MNPs crystallize in the magnetite phase (JCPDS No. 19-0629, Figure S1a) and exhibit a near-spherical morphology with an average diameter of approximately 10 nm (TEM, Figure S1b). Surface modification of the magnetite particles with citric acid results in the formation of a negative surface charge in a near-neutral aqueous medium, where the ζ-potential is −30 mV (LDE). This negative surface charge arises from the presence of deprotonated surface carboxyl groups of citric acid:

S-COOH + OH^−^ ⇌ S-COO^−^ + H_2_O (pH_IEP_ ~ 2.2, pK_a1_ = 3.95).

This strong surface charge provides high aggregative stability to the hydrosol, ensuring a uniform dispersion of the MNPs within the sodium alginate solution during the subsequent mixing step.

Dynamic light scattering (DLS) measurements of the citrate-modified MNPs in aqueous suspension gave a mean hydrodynamic diameter of 87.9 ± 0.8 nm and a polydispersity index of 0.206 ± 0.012, indicating a relatively narrow size distribution. The synthesis protocol involves ultrasonic dispersion of the precipitate until a constant mean hydrodynamic diameter is reached, thereby ensuring a reproducible state of aggregation. The significantly larger hydrodynamic diameter compared to the TEM size is primarily attributed to the formation of stable multi-particle aggregates (clusters), as well as the contribution of the hydration layer and the citrate shell surrounding these assemblies. DLS is highly sensitive to the presence of larger species, since scattering intensity scales with the sixth power of the particle radius. However, as demonstrated in our previous work [27], the citrate-modified MNPs are aggregatively stable over wide ranges of pH and electrolyte concentration, with electrosteric repulsion forces contributing to their increased stability.

**Figure 1:**
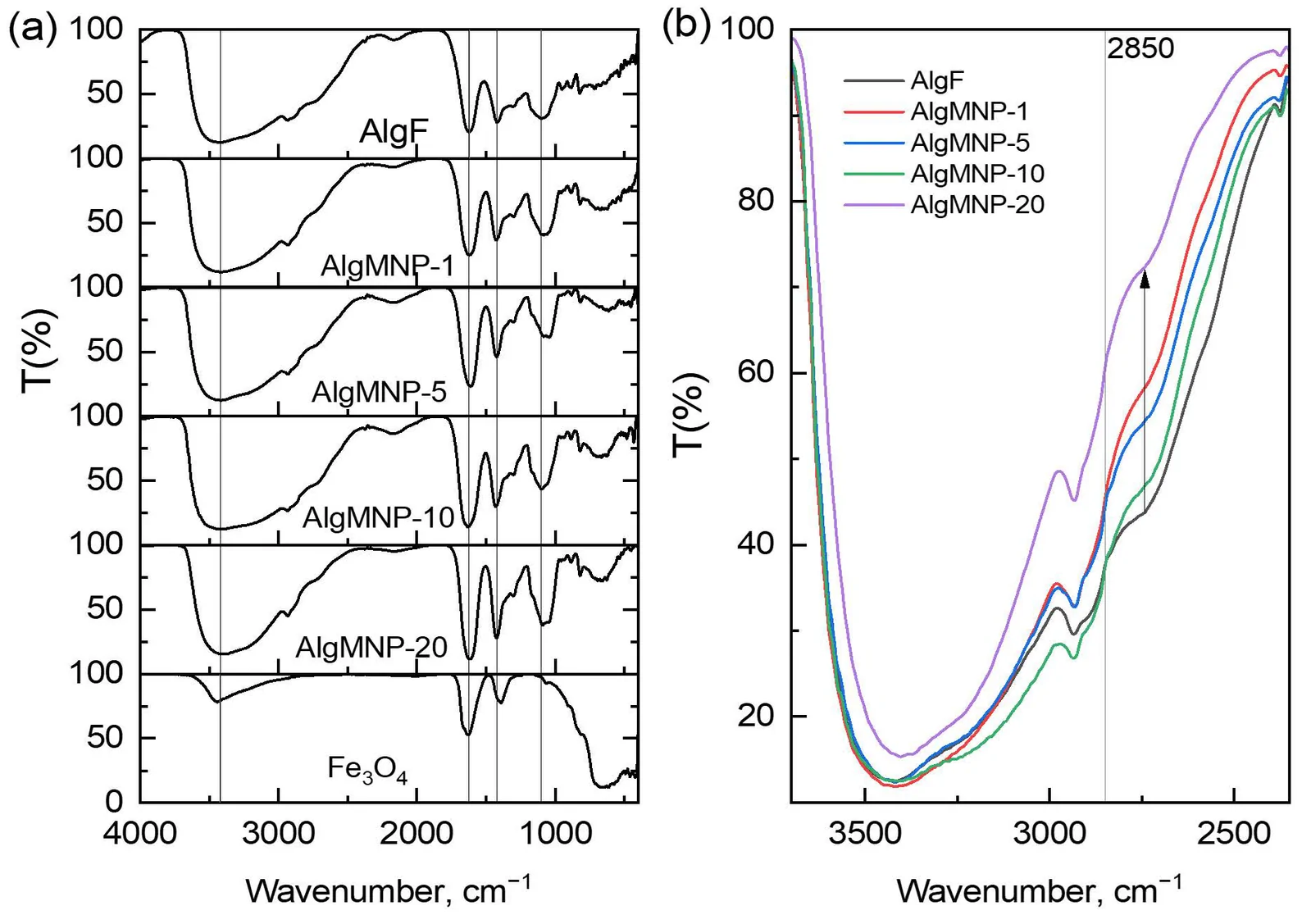
FTIR spectra of AlgF, MNPs and AlgMNP films: (**a**) survey spectra in the 4000–400 cm^−1^ range (AlgF reference bands are indicated: 3422, 1622, 1420 and 1101 cm^−1^); (**b**) magnified view of the high-wavenumber region (3700–2350 cm^−1^), where the arrow indicates the direction of decrease in the intensity of the ν(O–H) band in the low-frequency region (<2850 cm^−1^) when changing the concentration of MNPs.

The presence of the stabilizing agent is further confirmed by FTIR spectroscopy (Figure 1). In the FTIR spectrum of the modified MNPs, the intense bands at 1633 cm^−1^ (ν_as_(COO^−^)) and 1393 cm^−1^ (ν_s_(COO^−^)) confirm the presence of surface carboxylate groups. Additionally, the absence of a band near 1700 cm^−1^ is noted, indicating the lack of protonated carboxyl groups and confirming the chemical nature of adsorption (i.e., the absence of physically adsorbed acid molecules). The high intensity of these bands suggests a dense coating, and it can be assumed that the conditions used to obtain the particles (four washes with a concentrated (40 g/L) acid solution followed by washing with water) promote the coordination of citric acid with the surface through one or two carboxyl groups. Coordination involving three carboxyl groups is unlikely due to steric hindrance. In this case, one or two carboxylate groups remain free, oriented into the solution, thereby ensuring the stability of the sol. It is known that the difference in the positions of the bands, Δν = ν_as_ − ν_s_, allows a qualitative conclusion about the preferential binding mode of the carboxyl group COO to the oxide surface: Δν < 110 cm^−1^ indicates bridging bidentate coordination; Δν > 110 cm^−1^ indicates chelating bidentate coordination; and Δν > 200 cm^−1^ indicates covalent monodentate coordination [28]. In our case, Δν is 240 cm^−1^, which indicates monodentate binding, where a negatively charged oxygen of the carboxylate group forms a covalent bond with the metal ion.

The weak band at 1253 cm^−1^ arises from coupled C–O stretching and O–H deformation vibrations. Additionally, intense absorption bands at approximately 459 and 661 cm^−1^ indicate the formation of the Fe_3_O_4_ structure, characteristic of Fe–O vibrations. The weak band at 1063 cm^−1^ can be attributed to the C–O stretching vibrations of the hydroxyl group in citric acid.

Note that, in both Fe_3_O_4_ and AlgF, there are bands corresponding to ν_s_(COO^−^) and ν_as_(COO^−^), the positions of which differ slightly. The alginate matrix shows characteristic carboxylate bands at 1622 cm^−1^ (ν_as_(COO^−^)) and 1420 cm^−1^ (ν_s_(COO^−^)). Additional bands at 1300, 1101, and 1033 cm^−1^ correspond to C–C–H and C–O–C deformations and pyranose ring stretching. The C–O stretching and C1–H deformation bands in the 950–812 cm^−1^ region, typical of polysaccharides, indicate the presence of mannuronic and guluronic acid residues, while the 2900–3000 cm^−1^ bands are assigned to C–H stretching vibrations [29].

### 2.2. Structure and Physicochemical Properties of Composite Films

#### 2.2.1. Microstructure and Spectral Properties of Films

Figure S2 shows photographs of alginate films loaded with magnetite in both the dried and wet (hydrogel) states. The pure alginate film (AlgF) is the most transparent, whereas increasing the concentration of magnetite results in reduced visual transparency and a colour change from light brownish to black. The films exhibit a wavy surface, formed due to deformation during the drying process. The greatest deformation is observed in the AlgMNP-1 sample. Changes in the films’ transparency and color are associated with their spectral characteristics. The UV–visible transmission spectra of AlgF and AlgMNP films normalized to a reference thickness of 50 µm are presented in Figure S3. The spectrum of the original film displays a shoulder at 250 nm, which may be attributed to the presence of carbonyl groups in the alginate. Compared to the original alginate film, the incorporation of magnetite causes a significant decrease in normalized transmittance due to the strong absorption of UV and visible radiation by the magnetite particles. At a wavelength of 600 nm, the transmittance of the unloaded alginate film is approximately 70%, whereas for AlgMNP-1 it is about 26%, for AlgMNP-5 approximately 2.1%, and for AlgMNP-10-20 less than 1%.

The CIE LAB color parameters (L, a, b) of the films, derived from the transmission spectra, are summarized in Table S2 [30]. The AlgF film exhibits the highest lightness (L* = 85.6%), confirming its maximum transparency. A low positive b* value combined with an almost zero a* value indicates a slightly yellowish tint of the film. The addition of 1 wt% magnetite causes a sharp decrease in L* by 52.1%, reducing it to 33.5%. Positive values of a* (12.23) and b* (32.3) correspond to a brown color in this sample. Increasing the magnetite concentration to 5 wt% further decreases L* to 12.7%, while a* and b* remain positive, indicating a dark brown color. At magnetite concentrations of 10–20 wt%, L*, a*, and b* values approach zero, corresponding to a black color.

Thus, films containing 10 wt% MNPs or more are optically opaque. Due to their high UV-Vis absorption capacity, such materials can prevent photooxidative processes and be used as light-protective wound dressings, protecting sensitive skin or medicinal components from exposure to ultraviolet radiation [31]. While the opacity at 10 and 20 wt% MNPs provides effective light shielding, it may limit direct visual inspection of the wound bed. This trade-off could be addressed by using such materials as temporary protective layers for short therapeutic periods when photoprotection is prioritized.

**Figure 2:**
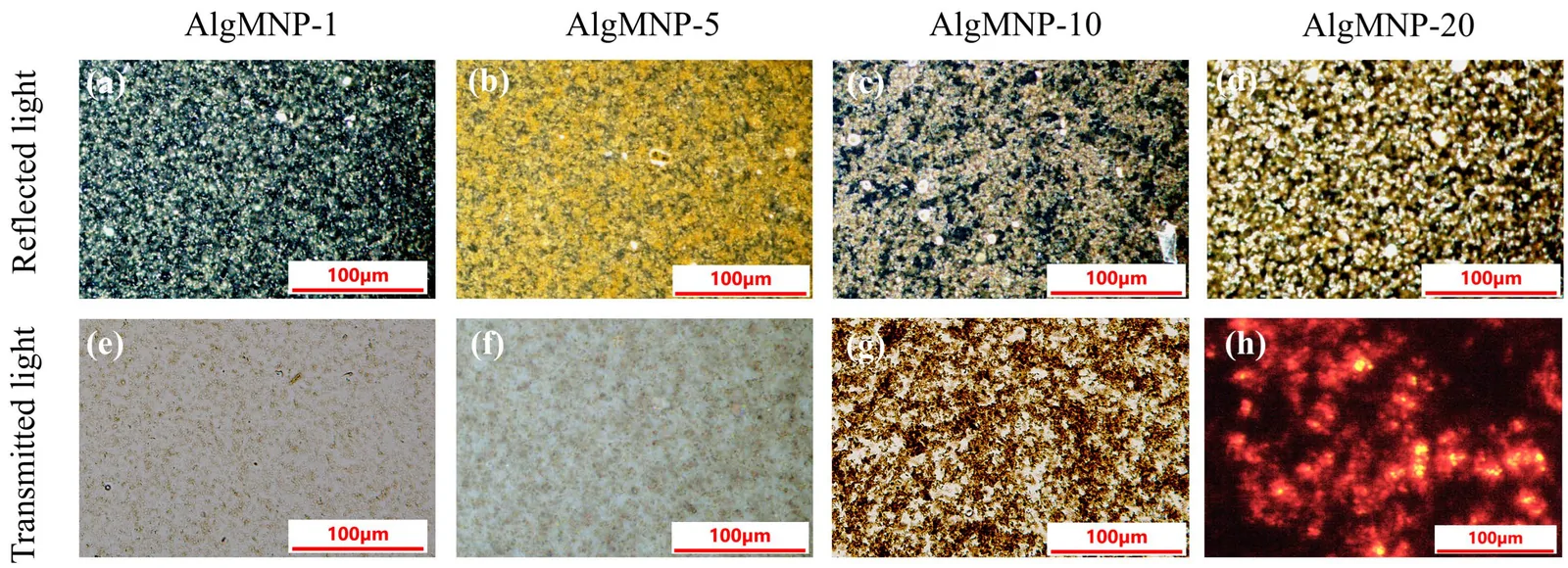
Optical micrographs of thin AlgMNP films on glass in various shooting modes: (**a**–**d**) reflected light microscopy mode; (**e**–**h**) transmitted light microscopy mode. Images (**a**,**e**) correspond to AlgMNP-1; (**b**,**f**)—AlgMNP-5; (**c**,**g**)—AlgMNP-10; (**d**,**h**)—AlgMNP-20. Scale bar 100 µm.

Figure 2 presents optical micrographs of thin AlgMNP films cast on a glass substrate, captured under various imaging modes, while Figure S4 provides complementary stereomicroscopy views of the thicker macro-films obtained in Petri dishes. At a low magnetite nanoparticle loading of 1 wt%, both sample types exhibit a highly uniform filler distribution within the polymer matrix. However, upon increasing the MNP concentration to 5 wt%, flocculation occurs despite the like-charged surfaces of both the magnetite nanocrystals and the film-forming polymer (sodium alginate matrix). This clustering is driven by the screened electrostatic repulsion at elevated ionic strength, which lowers the DLVO energy barrier and facilitates closer particle approaches. Notably, at this critical concentration (5 wt%), both the thin films on glass (Figure 2) and the thicker films from Petri dishes (Figure S4) consistently exhibit a branched network of MNP clusters, suggesting the possible formation of a continuous, interconnected morphology capable of acting as a robust percolation structure.

**Figure 3:**
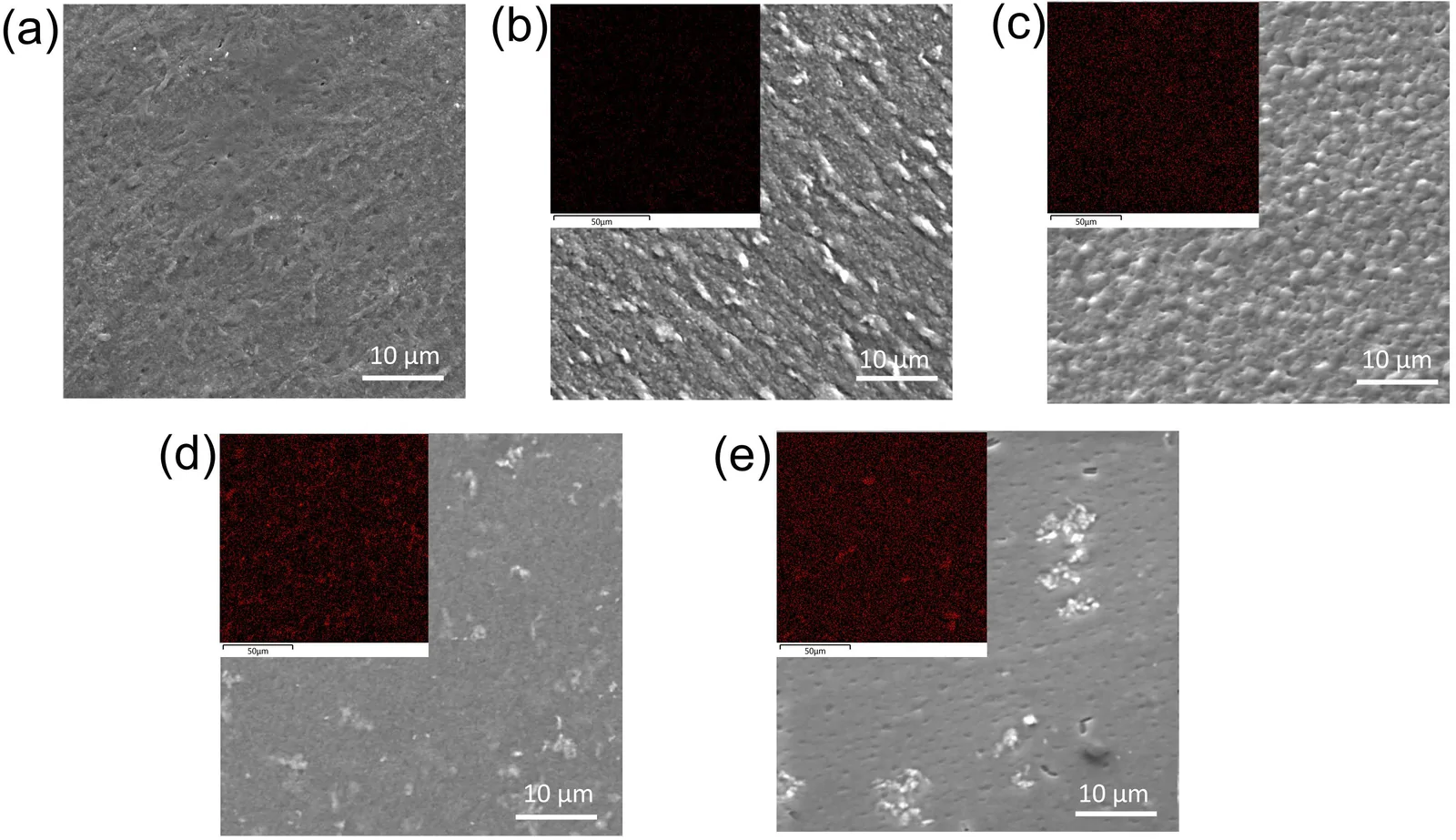
SEM micrographs (SE mode) and EDX mapping (Fe) for AlgMNP films. ((**a**)—AlgF, (**b**)—AlgMNP-1, (**c**)—AlgMNP-5, (**d**)—AlgMNP-10, (**e**)—AlgMNP-20.) SE imaging and EDX mapping confirm the presence of surface Fe aggregates at 10 and 20 wt% MNPs (**d**,**e**), while no surface aggregates are detected at 1 and 5 wt% (**b**,**c**). Scale bars: 10 µm.

Scanning electron microscopy of the sample surfaces revealed that the films of the original alginate possess a rough texture with raised structural elements of spherical and oblong shapes measuring 1–2 μm (Figure 3a). Additionally, there are numerous pores smaller than 1 micron in size. The incorporation of 1 wt% magnetite increased the film roughness and the frequency of structural inhomogeneities (Figure 3b).

When 5 wt% magnetite was added to the alginate matrix, the texture density increased while preserving structural heterogeneities of a globular shape, measuring 1–2 μm in size (Figure 3c). Imaging in different modes (SE for morphology assessment, BSE for phase contrast assessment) did not reveal the presence of magnetite on the film surface, indicating that the nanoparticles are distributed throughout the volume of the material.

The AlgMNP-10 film exhibits a dense structure and a smooth surface, on which magnetite aggregates are clearly visible (Figure 3d). The AlgMNP-20 film also has a dense structure and a smooth surface; however, depressions (possibly pores) measuring 0.5–1 μm in size are observed (Figure 3e). Magnetite aggregates are also present on the surface of this sample (Figure 3e and Figure S5a,c,e). In addition to the phase contrast, this observation is supported by energy-dispersive X-ray spectroscopy (EDX mapping) data, which show that the accumulation of magnetite aggregates corresponds to a denser distribution of iron atoms (Figure S5b,d,f).

In general, according to elemental mapping data, iron atoms are observed on the surface and in the near-surface layer of all films containing magnetite (Figure 3). An increase in Fe_3_O_4_ content corresponds to a higher distribution density of iron atoms in the EDX maps.

**Figure 4:**
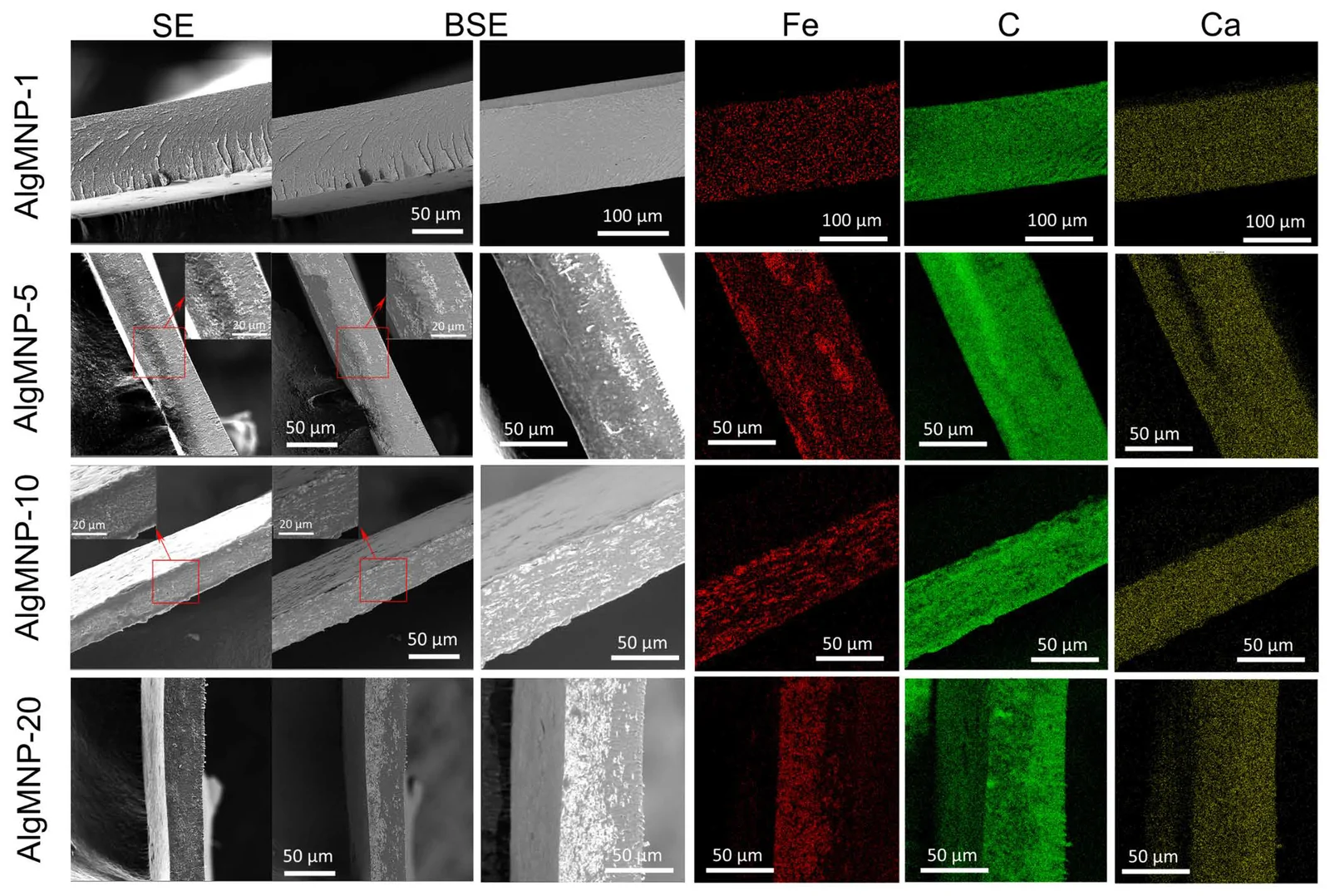
SEM micrographs in SE and BSE modes and EDX mapping of cross-sections of AlgMNP films.

To investigate the morphology of alginate films containing magnetite, their cross sections were analyzed (Figure 4). EDX mapping of the AlgMNP-1 sample revealed a homogeneous distribution of iron, carbon, calcium, and oxygen throughout the cross section, indicating uniform film composition. When the magnetite concentration increased to 5 wt%, clusters with a higher iron content (magnetite), oriented parallel to the film plane, were observed (Figure 4). This spatial arrangement is initiated during the stirring stage, where the high ionic strength of the polyelectrolyte medium compresses the electric double layer of the citrate-stabilized nanoparticles. Furthermore, partial adsorption of alginate chains onto the MNP surfaces can promote bridging flocculation, triggering the initial assembly into roughly isotropic cluster seeds. During the subsequent slow drying phase, as the film thins vertically, strong capillary forces and spatial confinement compress and deform these initial seeds, forcing them to spread laterally and reorganize into the elongated, in-plane oriented clusters seen in the cross-section (Figure 4). However, the concentration and density of such clusters on the cross-sectional surface were low, with the remaining iron distributed relatively evenly, consistent with observations of the surface microstructure. Quantitative analysis revealed that the clusters in the AlgMNP-5 sample had a mean length of 15.6 ± 2.6 µm and thickness of 4.4 ± 1.1 µm (Figure S6). The cluster dimensions represent apparent values measured on cross-sectional SEM images (Figure 4), while the true three-dimensional cluster sizes may differ.

At a magnetite content of 10 wt%, iron-enriched oriented clusters are observed more clearly; their concentration per unit area of the cut increases, while their size decreases. Thus, the mean cluster length was reduced to 4.8 ± 1.8 µm, and thickness to 1.0 ± 0.1 µm (*p* < 0.001 for both comparisons, Mann–Whitney U-test, Figure S6). At the same time, the iron concentration remains stable throughout the film thickness. According to the results of EDX mapping, areas deficient in carbon (and therefore with a reduced concentration of the polymer matrix) were identified, which may negatively affect the mechanical strength of the material.

In the case of a highly filled AlgMNP-20 sample, distinct stratification of the iron-containing phase is observed across the film thickness, characterized by MNP enrichment in the bottom region and depletion in the top region. This effect arises from the rapid gravitational sedimentation of large magnetite-containing aggregates at the early stages of the drying process, when the water content is high and the matrix viscosity is still low. The structural continuity of the matrix at the interface of these two layers is comprehensively confirmed by cross-sectional SEM and EDX elemental mapping (Figure 4). The EDX profiles demonstrate that while the Fe signal is predominantly concentrated in the lower zone of the film due to sedimentation, carbon (which forms the polysaccharide backbone of the polymer matrix) and calcium (the crosslinking agent) are present and continuously distributed throughout the entire cross-sectional thickness. Furthermore, SEM micrographs show a seamless interface completely free of microcracks, voids, or obvious delamination lines across the entire sample boundary. This provides direct evidence that the MNP aggregates settle through the liquid medium without disrupting the monolithic structure and integrity of the film. The resulting asymmetrical distribution of the filler yields a monolithic material with a well-defined two-layer structure, wherein both layers are interconnected through the continuous alginate matrix, thus exhibiting pronounced structural anisotropy.

It has been established that the dependence of the dry-state film thickness on the concentration of magnetite nanoparticles exhibits a non-monotonic behavior. The thickness of the initial alginate film is 48 μm (Figure S7). The incorporation of 1 wt% magnetite results in a significant increase in film thickness to 80 μm. This initial thickening can be attributed to a loosening of the macromolecular packing, resulting from the disruption of the original alginate matrix. The introduction of similarly charged MNPs into the polymer solution restricts the tight alignment of macromolecules, thereby weakening hydrogen bonds and disturbing the subsequent development of the ionically crosslinked egg-box network, as supported by FTIR data (see below). Conversely, increasing the nanoparticle content to 5 and 10 wt% causes the average thickness to contract back to 48 μm and then decrease to 36 μm, respectively (Figure 4). This thinning is associated with the compaction of the polymer matrix driven by filler-induced densification and the excluded volume effect of MNP clusters. A further increase in magnetite concentration to 20 wt% causes no significant change in the sample thickness, indicating that a limit of structural compaction has been reached.

When considering the filled composite films, it was noted that their FTIR spectra closely resemble those of the original alginate matrix (Figure 1). The absence of new bands or significant shifts in the FTIR spectra of the composite films, even at high MNP loadings, indicates the lack of strong ionic interactions between alginate carboxylates and the citrate-modified MNP surface. Since the MNPs are introduced before Ca^2+^ crosslinking, their negative charge promotes electrostatic repulsion from the alginate chains, which disrupts chain packing and weakens the polymer network upon subsequent crosslinking. Unlike AlgF, a more pronounced group of bands at 450–750 cm^−1^ is observed in the spectra of AlgMNP films, which is directly attributed to the Fe–O stretching vibrations of the magnetic nanoparticles. Additionally, in contrast to MNPs, the spectra of AlgF and AlgMNP films exhibit a strongly broadened band of stretching vibrations of OH groups, with a maximum at approximately 3400 cm^−1^, characteristic of alginate materials. This broadening is influenced by the disorder within the network of intra- and intermolecular hydrogen bonds. The introduction of magnetite nanoparticles generally leads to a decrease in the intensity of the band in the low-frequency region (<2850 cm^−1^), which corresponds to strong hydrogen bonds, while the intensity of the band in the high-frequency region (3300–3600 cm^−1^) is maintained. This effect is attributed to extension and “loosening” of intermolecular hydrogen bonds within the alginate matrix upon the addition of similarly charged MNPs into the interchain spaces of alginate during gel formation. It is noteworthy that the strongest decrease in intensity in the low-frequency region ν(O-H), and hence a stronger decrease in the contribution of hydrogen bonds, is observed for the sample with the lowest MNP concentration (AlgMNP-1), which confirms the strong loosening of hydrogen bonds at a high degree of MNP penetration into the interchain spaces of alginate. For AlgMNP-20, a decrease in the intensity of the entire ν(O-H) band is observed in both the high-frequency and low-frequency regions.

#### 2.2.2. Simultaneous Thermal Analysis

**Figure 5:**
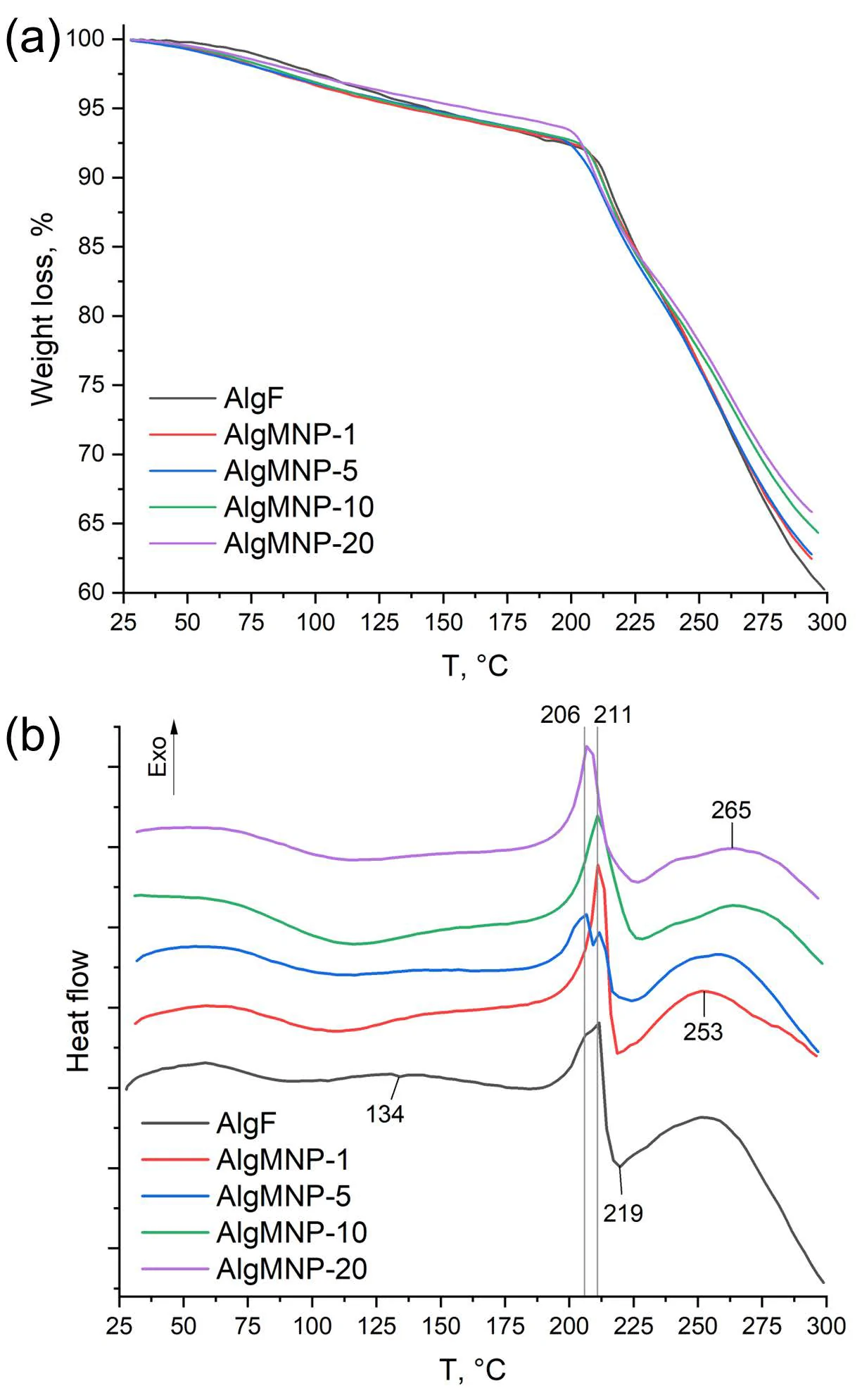
Thermogravimetric analysis (**a**) and differential scanning calorimetry data (**b**) for sodium alginate films and their modified Fe_3_O_4_ derivatives.

At temperatures up to 300 °C in an inert atmosphere, mass losses in the alginate/Fe_3_O_4_ system can be attributed to the loss of free and bound water molecules [32] and thermolysis processes accompanied by decarboxylation, decarbonylation, and dehydration of sodium alginate [33], as well as the decomposition of citric acid, which stabilizes magnetite nanoparticles through their sequential conversion to aconitic acid (175 °C) and itaconic acid (190–210 °C) (Figure 5a) [34]. Up to 190 °C, all mass losses are associated with the removal of water molecules adsorbed within the hydrophilic polymer matrix. The smooth, gradual profile of the mass decrease, without abrupt changes, suggests that there are practically no free water molecules present in the film [32].

Figure 5b presents the differential scanning calorimetry data. Within the temperature range of 25–60 °C, a mild exothermic effect is observed, attributed to structural rearrangements in the alginate polymer matrix, resulting in the formation of more kinetically favourable supramolecular structures [35]. In the higher temperature region (190–220 °C), an intense exothermic peak appears in the DSC curves of the composites, which coincides with a sharp weight loss in the TGA profile (Figure 5a). This corresponds to the main depolymerization stage of sodium alginate, which involves dehydration of the polysaccharide chains, cleavage of glycosidic bonds, decarboxylation, and the initial formation of a carbonaceous residue.

With an increase in the mass fraction of magnetite, the effect of these structural rearrangements diminishes, which may be linked to a more stable new spatial orientation of the alginate polyelectrolyte molecules relative to the Fe_3_O_4_ nanoparticles. The formation of these new structures is further evidenced by the disappearance of the endothermic peak characteristic of the glass transition temperature of sodium alginate (134 °C) [36], the absence of the endothermic melting peak of sodium alginate at 215–220 °C in samples containing 10–20 wt% Fe_3_O_4_, and a shift in the onset temperature of pyrolysis of the organic component with increasing magnetite content from 253 °C (AlgF) to 265 °C (AlgMNP-20).

Several explanations can be proposed for this process observed upon the introduction of MNPs stabilized with citric acid. Literature reports indicate that in sodium alginate films, Ca^2+^ ions bind approximately 40% of COONa groups [37]. The carboxyl group of citric acid, possessing more pronounced acidic properties (pK_a1_ = 3.1) [38] compared to sodium alginate (pK_a_ = 3.5) [39,40], can interact with calcium ions, disrupting intermolecular crosslinks within the original gel structure. It is also important to consider that alginate is a pH-sensitive polymer; the introduction of magnetite nanoparticles stabilized with citric acid locally shifts the pH towards a more acidic environment, suppressing the dissociation of the carboxyl groups of the polyelectrolyte molecule and consequently reducing the number of active carboxyl groups in the alginate molecule near the nanoparticle. This effect may further contribute to the disruption of intermolecular crosslinks in the sodium alginate film by Ca^2+^ ions. Collectively, these processes can account for the absence of the glass transition temperature (130–135 °C) and the pronounced melting temperature (210–220 °C) characteristic of sodium alginate films [36] in samples containing magnetite nanoparticles. These findings are corroborated by FTIR spectroscopy, which revealed a weakening of hydrogen bonds upon the incorporation of magnetite.

At a nanoparticle mass fraction of 5 wt%, an optimum balance is achieved between the formation of supramolecular structures cross-linked with calcium ions and those modified with magnetite, resulting in the appearance of two peaks on the DSC curve within the temperature range of 190–220 °C. In AlgMNP films, the attenuation of the sodium alginate melting peak at 219 °C may also be attributed to the enhanced decomposition of the citric acid thermolysis products, as described above, with increasing Fe_3_O_4_ mass fraction.

Thus, the modification of sodium alginate films with magnetite nanoparticles involves the acid–base interaction of citric acid, which imparts a negative charge to the Fe_3_O_4_ surface, with Ca^2+^ ions that crosslink the alginate structure. Increasing the concentration of magnetite nanoparticles results in the gradual replacement of the original structure—formed through intermolecular interactions between the carboxyl groups of alginate and Ca^2+^ ions—with a magnetite-modified structure. Ultimately, the disappearance of thermal transitions in the AlgMNP composites reflects a complex network rearrangement, driven both by initial electrostatic repulsion between the components and the subsequent competitive binding of Ca^2+^ ions by citrate groups, rather than the formation of additional crosslinks.

#### 2.2.3. Analysis of Mechanical Properties

**Figure 6:**
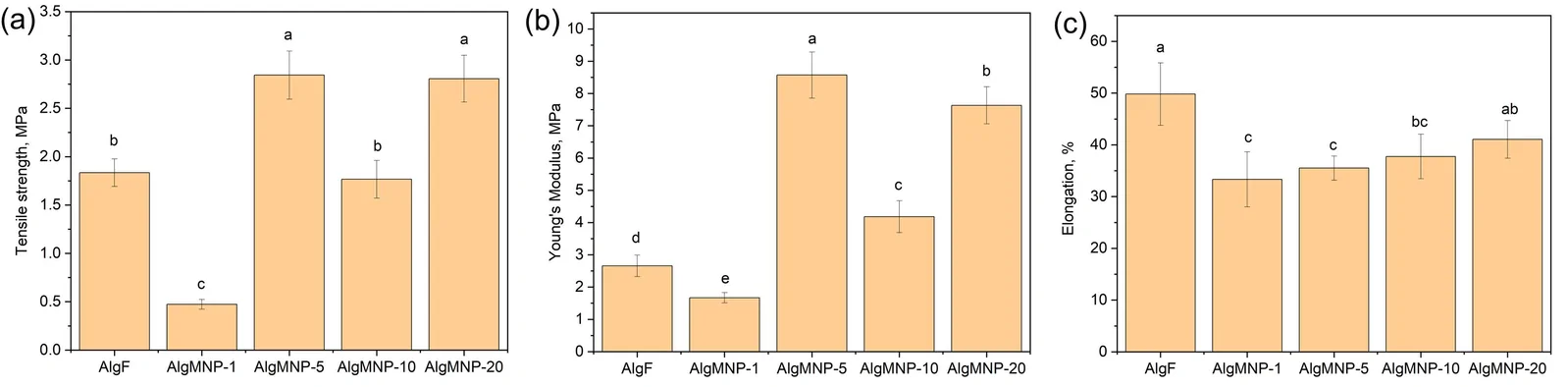
Mechanical properties of alginate composite films with different MNP concentrations: (**a**) tensile strength, (**b**) Young’s modulus, (**c**) elongation at break. Data are presented as mean ± SD (*n* > 5). Different letters above bars indicate statistically significant differences between groups (Kruskal–Wallis test followed by Conover’s post hoc test, *p* < 0.05). Groups sharing the same letter are not significantly different. Letters are assigned in descending order of mean values (a—highest).

The observed changes in the strength characteristics of alginate hydrogels pre-swollen in Hanks solution at pH 5.5, with increasing concentrations of like-charged magnetite nanoparticles, demonstrate a complex, non-monotonic dependence resulting from alterations in their microstructure (Figure 6). The tensile strength (*σ*) and Young’s modulus (*E*) generally follow similar trends (Figure 6a,b). For the unfilled AlgF hydrogel, the mechanical properties (*σ* = 1.83 ± 0.14 MPa, *E* = 2.66 ± 0.33 MPa, *ε* = 47 ± 6%) are characteristic of a hydrated biopolymer network. The presence of water acts as a plasticizer, screening electrostatic interactions between alginate chains and weakening hydrogen bonds, which reduces intermolecular forces. This plasticization leads to a decrease in *σ* and *E* compared to dry alginate films, while simultaneously increasing elongation at break due to enhanced chain mobility.

Compared to the initial alginate film, the introduction of 1 wt% Fe_3_O_4_ causes a significant decrease in *σ* (0.47 ± 0.05 MPa, *p* < 0.001), *E* (1.67 ± 0.16 MPa, *p* < 0.001), and *ε* (33 ± 5%, *p* < 0.001) to minimum values within the studied concentration range. This effect may be attributed to the uniform dispersion of nanoparticles bearing the same charge as the polymer matrix (Figure 2a,e and Figure 3b), which facilitates their incorporation into the interchain spaces of the alginate. Such distribution leads to partial disruption of the original polymer network structure due to the weakening of hydrogen bonds and ionic crosslinks, as supported by FTIR data, but does not yet result in effective reinforcement, thereby reducing the mechanical strength of the hydrogel (Figure 6). The weakening of the structure is also confirmed by a noticeable increase in the thickness of dry films from 48 µm (AlgF) to 80 µm (AlgMNP-1) (Figure 4), indicating an expansion of the polymer matrix.

A further increase in magnetite concentration to 5 wt% resulted in a sharp rise in both tensile strength (*σ* = 2.84 ± 0.25 MPa) and Young’s modulus (*E* = 8.6 ± 0.7 MPa), reaching the highest values among all samples (for *σ*, the AlgMNP-5 and AlgMNP-20 samples (both group a) were not significantly different from each other (*p* > 0.05), but *E* was significantly higher than other samples (*p* < 0.001)). The elongation at break remained relatively low (*ε* = 35.5 ± 2.3%) and was not significantly different from the AlgMNP-1 sample (*p* > 0.05). This suggests the formation of a material with increased rigidity and brittleness. As demonstrated above, flocculation of magnetite nanoparticles occurs in this system (Figure 2 and Figure S4), while maintaining a relatively uniform distribution of iron and carbon throughout the sample (Figure 3c and Figure 4). Additionally, a significant reduction in film thickness to 48 μm is observed, indicating substantial matrix densification. Quantitative SEM analysis revealed the formation of elongated clusters with a mean length of 15.6 ± 2.6 µm and thickness of 4.4 ± 1.1 µm. These clusters, as seen in optical micrographs (Figure S4), appear to form a continuous, interconnected, branching reinforcing structure (percolation network) that compacts and strengthens the matrix through the excluded volume effect, while residual uniformly distributed nanoparticles restrict the mobility of polymer chains, limiting plastic deformation.

With the introduction of 10 wt% magnetite, both the strength and Young’s modulus decrease sharply (*p* < 0.001) to *σ* = 1.77 ± 0.20 MPa and *E* = 4.2 ± 0.5 MPa, respectively, approaching the levels observed in the pristine AlgF alginate film. Specifically, the *σ* value of AlgMNP-10 showed no statistically significant difference from AlgF (*p* > 0.05), whereas its *E* value remained significantly higher (*p* < 0.001). However, the elongation at break exhibited a slight, statistically insignificant increase to 37.7 ± 4.3%. The decrease in mechanical properties at 10 wt% MNPs arises from a combined mechanism involving localized network disruption and structural stress concentration. DSC data (Section 2.2.2) show that at this loading, the characteristic melting and glass transition peaks of alginate disappear, indicating disruption of the egg-box network due to competitive binding of Ca^2+^ ions by citrate groups on the MNP surface. Additionally, quantitative SEM analysis (Figure S6) reveals that the clusters at 10 wt% are significantly smaller (4.8 µm) and denser than at 5 wt% (15.6 µm). EDX mapping (Figure 4) confirms that these dense aggregates remain well isolated rather than forming a continuous network. Under tensile load, these isolated rigid clusters act as internal stress concentrators, promoting premature crack initiation and propagation through the altered polymer network.

Compared to AlgMNP-10, the highly filled AlgMNP-20 sample exhibits a significant (*p* < 0.001) increase in both the strength (*σ* = 2.81 ± 0.24 MPa) and Young’s modulus (*E* = 7.6 ± 0.6 MPa). The elongation at break remains high (*ε* = 41.1 ± 3.6%), showing no significant change compared to AlgF and AlgMNP-10 (*p* > 0.05). The combination of high strength and rigidity with preserved elasticity can be attributed to the apparent stratification of the iron-containing phase throughout the film thickness, resulting from sedimentation of large magnetite clusters during the slow hydrogel drying. Morphologically, the AlgMNP-20 sample can be considered as a superposition of two integrated alginate matrices: the lower layer heavily saturated with MNP aggregates, and the upper polymer-rich layer depleted of them (Figure 4). Despite this apparent compositional gradient, the sample does not undergo thermodynamic phase separation or mechanical delamination. Because both parts are derived from the same initial hydrogel precursor, the alginate matrix is expected to maintain chain interlacing throughout the sample thickness, including the interface. This is assumed to ensure robust interfacial adhesion, enabling effective stress transfer and preventing delamination under tensile load. Based on the observed stratification, it can be proposed that when a microcrack initiates in the brittle, highly filled bottom layer, its propagation may be arrested as it enters the compliant, elastic upper layer. This crack-arrest mechanism, while speculative, is consistent with the observed combination of high strength and preserved ductility.

The results obtained suggest the possibility of controlling the mechanical properties of hydrogels—including strength, rigidity, and elasticity—by regulating the microstructure of the films through variation in the concentration of the magnetic filler.

#### 2.2.4. Wettability Analysis (Contact Angle Measurement)

The contact angles of alginate films with diiodomethane, water, glycerol, ethylene glycol, and formamide, each with varying MNP content, as well as the calculated γ_s_^LW^, γ_s_^+^, and γ_s_^−^ values, are presented in Table S3 and Figure S8. It is evident that γ_s_^LW^ significantly decreases only for AlgMNP-1, which we attribute to the increasing contribution of donor–acceptor interactions between the MNPs and the alginate matrix, alongside a decreasing contribution from nonpolar interactions.

The electron-donor component γ_s_^−^ increases with rising MNP content, from 24.6 mJ/m^2^ (AlgF) to 61.9 mJ/m^2^ (AlgMNP-20). This increase is attributed to the presence of surface citrate anions on the MNPs, which contain three carboxylate groups exhibiting a high γ^−^ value (up to 55–60 mJ/m^2^ for pure sodium citrate) [41].

The electron-accepting component γ_s_^+^ remains extremely low (0.2–1.7 mJ/m^2^) for all samples, which is typical of highly hydrophilic polysaccharide and oxide surfaces that are virtually devoid of electron-accepting groups in neutral and slightly alkaline environments.

The maximum value of the total free surface energy, γ_s_^TOT^ = γ_s_^LW^ + 2(γ_s_^+^·γ_s_^−^)^0.5^, is achieved at 20 wt% MNPs (approximately 68 mJ/m^2^). This corresponds with the minimum water contact angle (θH_2_O ≈ 16°) and confirms the most pronounced hydrophilic character of the surface at this composition.

#### 2.2.5. Magnetic Response

To evaluate the macroscopic magnetic response of the hydrogel biomaterials, an attraction test using a permanent NdFeB magnet (class N52, diameter 50 mm, thickness 30 mm) was performed (Figure S9). The experimental procedure is described in detail in the Supporting Information. All MNP-containing composites exhibited clear attraction to the magnet. The trigger distance for visible displacement expectedly increased with the MNP loading, from 10 mm for the AlgMNP-1 sample to 65 mm for the AlgMNP-20 sample. This trend reflects the higher volume fraction of the magnetic phase localized within the polymer matrix.

### 2.3. Results of Biological Studies

#### 2.3.1. Evaluation of the Biocompatibility of Hydrogel Biomaterials with Human Fibroblasts In Vitro

**Figure 7:**
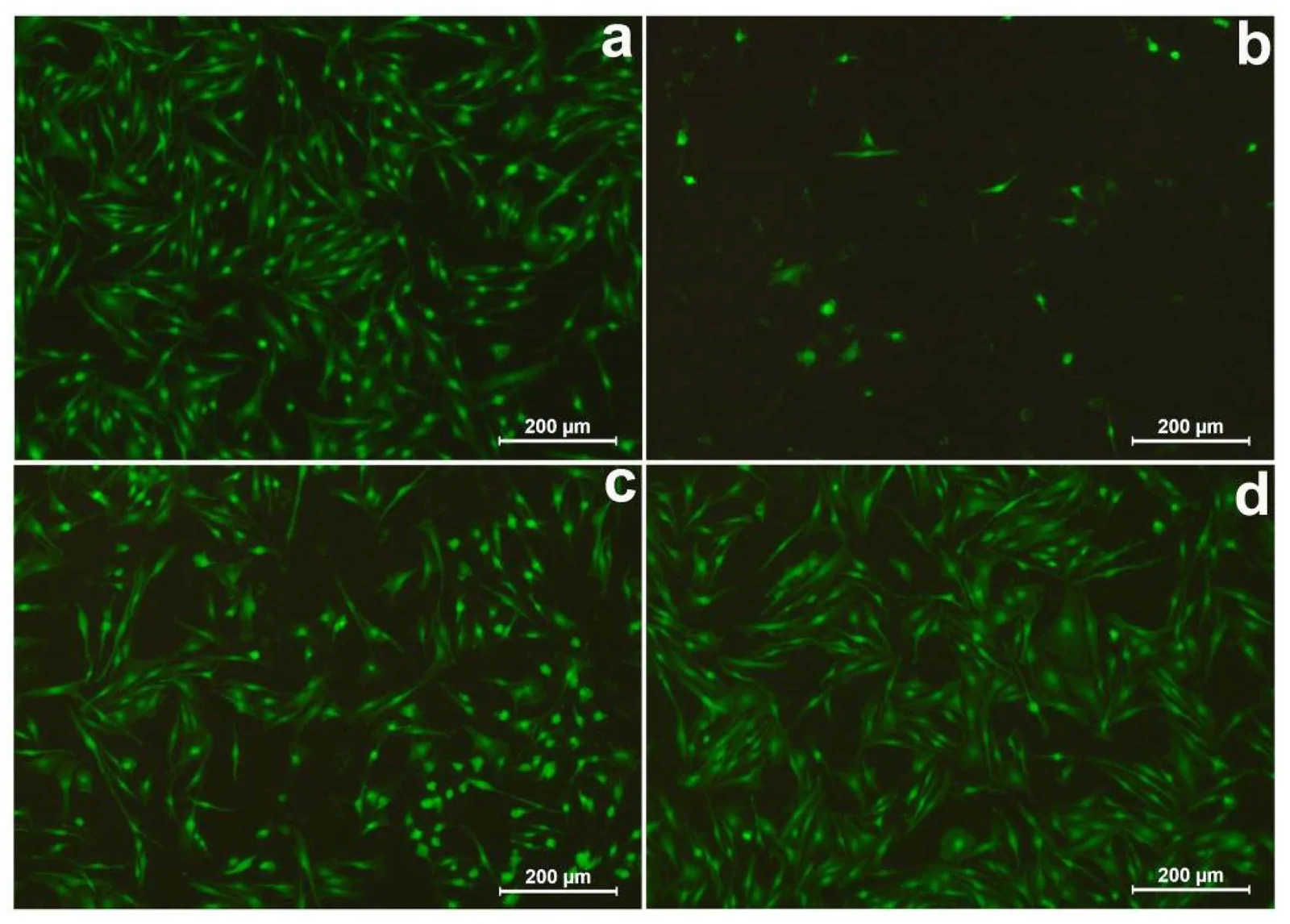
Human fibroblasts after 24 h of co-culture with hydrogel materials. Note: (**a**)—control, (**b**)—AlgF, (**c**)—AlgMNP-10, (**d**)—AlgMNP-20. Note: magnification 50×, scale bar 200 µm, stained with Calcein AM.

*Morphometric characterization of fibroblasts during co-incubation with hydrogel films.* In the control group, after 24 h of cultivation, all fibroblasts exhibited a spindle-shaped morphology, with cell length, including filopodia, measuring 73 ± 16 μm (Figure 7a and Figure S7). It was observed that the addition of AlgF to the culture medium delayed the transformation and growth of fibroblasts; most cells retained a near-spherical shape, and the length of spindle-shaped cells was 48 ± 6 μm (Figure 7b and Figure S10). Furthermore, it should be noted that when cells were treated with calcein acetoxymethyl ester (Calcein AM), a significant proportion of cells exhibited reduced metabolic activity (Figure 7b). When fibroblasts were incubated with AlgMNP-10 and AlgMNP-20, their metabolic activity and morphometric characteristics were comparable to control values, with cell lengths of 78 ± 13 μm and 81 ± 15 μm, respectively (Figure 7c,d and Figure S10).

As a rule, a delay in cell transformation may indicate a reduction in cell proliferative activity. Therefore, the proliferative activity of fibroblasts was assessed during co-culture with hydrogel films over a 48 h period.

*Assessment of the proliferative and metabolic activity of fibroblasts during co-incubation with hydrogel films.* When assessing fibroblast proliferative activity, the cell monolayer density six hours after adding the cell suspension to the wells of the culture plates was considered 100%. The cell monolayer density at the initial observation point was comparable across all groups, averaging 33 ± 3 cells per 200 μm^2^.

**Figure 8:**
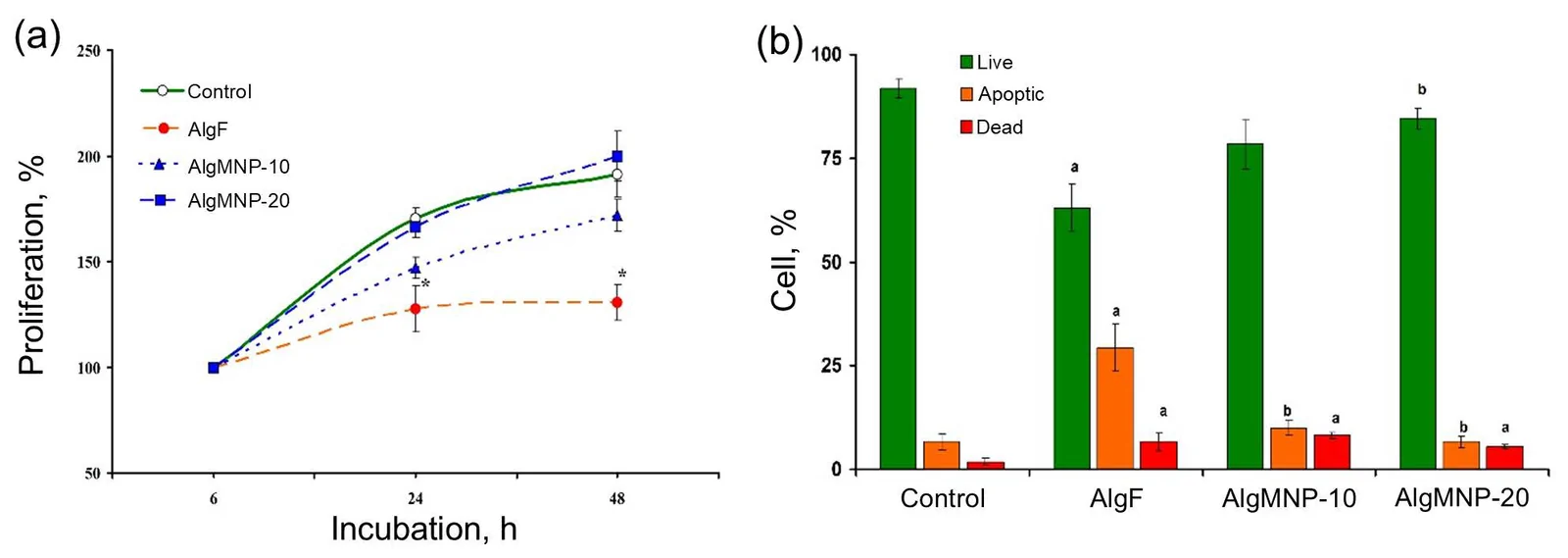
Fibroblast growth curve during incubation with AlgF, AlgMNP-10 and AlgMNP-20 hydrogel materials (**a**); Effect of hydrogel materials on viability of fibroblasts after 48 h of co-incubation (**b**). Data are expressed as Mean ± SD; ^a^—differences are significant compared to Control, at *p* < 0.05, ^b^—differences are significant compared to AlgF, at *p* < 0.05; *n* = 5. * *p* < 0.05—reliability of differences with readings after blood incubation with the control without film.

In the control group, after 48 h of cultivation, the number of fibroblasts doubled, reaching 65 ± 7 cells per 200 μm^2^ (Figure 8a). The addition of AlgF to the culture medium reduced the proliferative activity of fibroblasts: after 48 h of incubation, the cell count was 47 ± 4 cells per 200 μm^2^, which is comparable to the initial monolayer density of 36 ± 7 cells per 200 μm^2^ (Figure 8a).

When fibroblasts are incubated with AlgMNP-10, their proliferative activity slows during the first day of culture but recovers by the second day (Figure 8a). Fibroblasts cultured in the presence of AlgMNP-20 exhibit proliferative activity comparable to that of the control (Figure 8a). Thus, it was demonstrated that increasing the MNP content in the alginate matrix enhances the cytocompatibility of the biomaterial.

The results of the morphometric characterization of cellular responses to the studied materials are consistent with those of the assessment of the numbers of live, apoptotic, and dead cells obtained using flow cytometry.

In the control, when fibroblasts were incubated under standard culture conditions, the proportions of live, apoptotic, and dead cells in the population were 92 ± 2%, 7 ± 2%, and 2 ± 1%, respectively (Figure 8b). Co-incubation of fibroblasts with AlgF increased the proportions of apoptotic and dead cells in the population to 29 ± 6% and 7 ± 2%, respectively (Figure 8b).

The observed increase in cell viability at higher MNP content is consistent with our recent independent study on human dermal fibroblasts [42], which demonstrated that citrate-modified MNPs do not exert cytotoxic effects at concentrations below 1.0 mg/mL. When embedded in the alginate matrix, the local concentration of accessible iron remains well below this safe threshold, as the polymer network restricts nanoparticle release. This is further supported by the hemolysis data (Section 2.3.2, Table S4), which show no signs of MNP-induced oxidative damage to erythrocyte membranes during 2 h of incubation.

Thus, the addition of MNPs to the film enhances the biocompatibility of the material, leading to an increase in the number of viable cells and a reduction in the number of apoptotic cells to a level comparable with the control (Figure 8b).

#### 2.3.2. Analysis of the Effects of Hydrogel Materials and Their Components on Blood and Plasma Coagulation In Vitro

Ghaffari Sharaf et al. [43] employed the BRT test to evaluate the hemocompatibility of cyclodextrin-modified magnetic nanoparticle-based adsorbents. In this study, to assess the hemocompatibility of hydrogel biomaterials in vitro, we examined their effects on blood coagulation using the BRT test [44], on the coagulation of platelet-poor plasma using the APTT test [45], and on erythrocyte hemolysis [46].

Figure 8a illustrates a significant reduction in coagulation time in the BRT test following 10 or 20 min of incubation with AlgF films (S_1_: 0.5 cm × 0.5 cm = 0.25 cm^2^ and S_2_: 0.75 cm × 0.75 cm = 0.56 cm^2^) and AlgMNP films (S_1_ and S_2_), compared to the control without film. The reduction ranged from 1.7 to 14.8 times for AlgF films and from 2.61 to 39.1 times for AlgMNP films, respectively. The potential advantage of the procoagulant activity of AlgMNP-5, AlgMNP-10, and AlgMNP-20 films after 10 min of incubation, and AlgMNP-5 and AlgMNP-10 films after 20 min of incubation, compared to AlgF films, was found to be significant only in experiments involving films of area S_1_.

Thus, incubation of blood with AlgMNP films results in a significant reduction in coagulation time—up to 40-fold compared to the control without film—in the BRT test, depending on the film area and incubation time.

**Figure 9:**
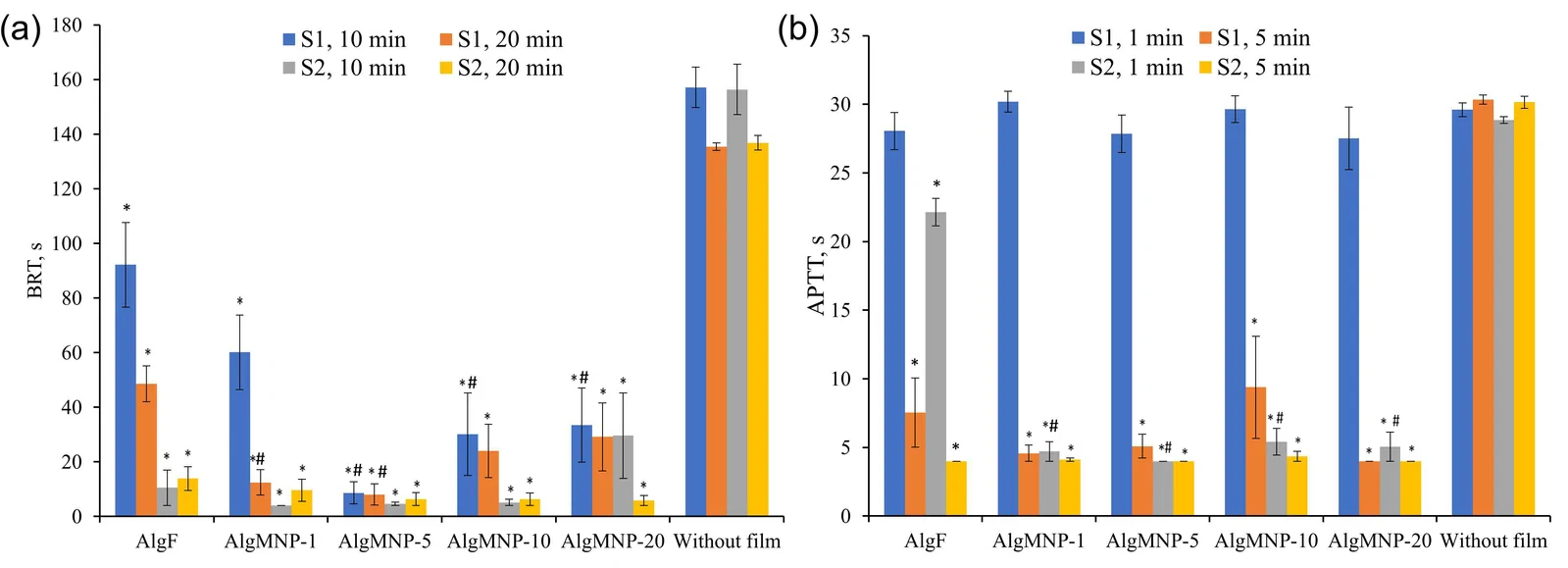
Blood clotting time (**a**) in the BRT test after incubation with AlgF or AlgMNP. Without film—controls without films after 10 or 20 min of blood incubation at 37 °C; blood clotting time in the controls without films before incubation—195.99 ± 16.58 s (for group S_1_, 10 min), 195.14 ± 13.57 s (for group S_1_, 20 min), 158.1 ± 9.21 s (for group S_2_, 10 min), 157.78 ± 10.05 s (for group S_2_, 20 min); * *p* < 0.05—reliability of differences with readings after blood incubation with the control without film, # *p* < 0.05—reliability of differences with readings after blood incubation with AlgF; *n* = 5–6. Plasma clotting time (**b**) in the APTT test after incubation with AlgF or AlgMNP. Without film—controls without films after 1 min or 5 min of plasma incubation at 37 °C; plasma clotting time in controls without films before incubation was 29.38 ± 0.52 s (for group S_1_, 1 min), 30.4 ± 0.52 s (for group S_1_, 5 min), 27.89 ± 0.24 s (for group S_2_, 1 min), 29.88 ± 0.43 s (for group S_2_, 5 min); * *p* < 0.05—significance of differences with the control without film; # *p* < 0.05—significance of differences after plasma incubation with AlgF; *n* = 5.

Figure 9b illustrates the effect of incubation with AlgF and AlgMNP films on plasma coagulation in the APTT test. After 5 min of incubation, plasma exposed to AlgF and AlgMNP films of S_1_ area exhibited a significant reduction in coagulation time compared to the control without film, by factors of 4.02 and 3.23–7.59, respectively. Following 1 min and 5 min incubation of S_2_-area samples, a significant decrease in plasma coagulation time was observed relative to the control without film: 1.3-fold for AlgF and 5.33–9.71-fold for AlgMNP after 1 min, and 7.54-fold for AlgF and 6.92–7.54-fold for AlgMNP after 5 min. Incubation of plasma with S_2_-area AlgMNP films for 1 min resulted in a significantly greater reduction in coagulation time compared to incubation with AlgF films.

Thus, incubation of plasma with AlgMNP films results in a significant reduction in coagulation time—up to 9.7-fold compared to the control without film—in the APTT test, depending on the film area and incubation time.

Table S4 shows the effect of incubating the films in the erythrocyte suspension on the optical density of the supernatant (ODS) obtained after centrifugation. No significant differences were observed between the ODS in the buffer control and the ODS after incubation with AlgMNP composite films of any size. The degree of erythrocyte hemolysis (DEH) after incubation with AlgF S_1_ and S_2_ films was 0.90 ± 0.37% and 0.59 ± 0.59%, respectively. DEH after incubation with AlgMNP S_1_ and S_2_ films ranged from 0.26 ± 0.15% to 1.30 ± 0.61% and from 0 ± 0% to 1.08 ± 0.62%, respectively. However, no significant differences were observed between the erythrocyte DEH readings after incubation with the original sample and after incubation with AlgMNP composite films. Medina-Moreno A. et al. [47] showed that the DEH after incubation with magnetite/chitosan nanoparticles was approximately 2%.

An in vitro hemocompatibility assessment of Fe_3_O_4_-filled alginate hydrogels demonstrated that these biomaterials reduce blood and plasma coagulation times, indicating a procoagulant effect. This effect increases with larger film area and longer incubation periods. The degree of red blood cell hemolysis upon contact with the films does not exceed 2%, classifying the materials as non-hemolytic. Consequently, these systems may be utilized to develop materials with wound-healing properties.

### 2.4. Correlation of Microstructure and Functional Properties

**Figure 10:**
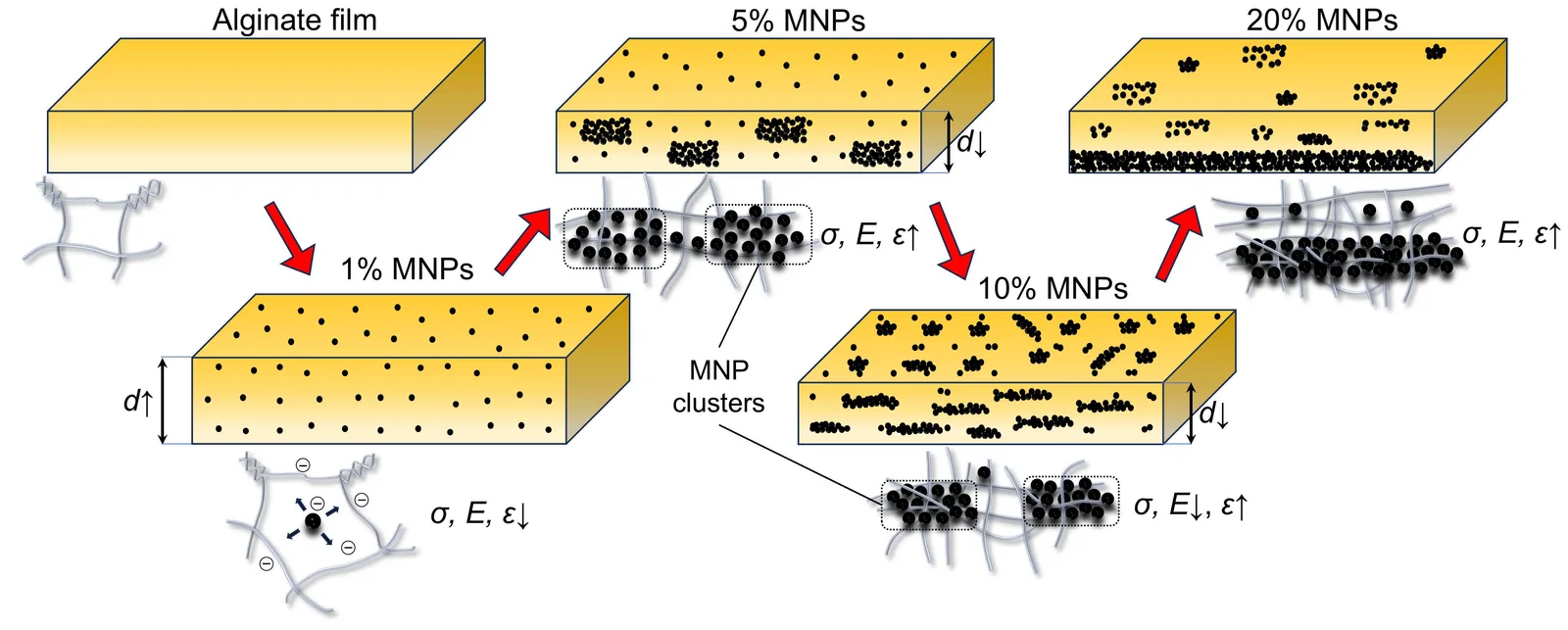
Schematic illustration of the concentration-dependent evolution of microstructure and its impact on the film thickness (*d*) and mechanical properties (*σ*, *E*, *ε*) of alginate-based composites filled with MNPs. The arrows indicate the direction of changes (growth or decrease) in mechanical parameters and film thickness relative to the preceding sample in the sequence of increasing magnetite concentration.

It has been established that increasing the concentration of magnetic nanoparticles induces changes in the microstructure of materials, thereby affecting their mechanical, thermal, and biological properties. Figure 10 presents a summary model illustrating the microstructure formation of alginate hydrogels as the magnetite nanoparticle content increases. The initial alginate matrix is characterized by an egg-box structure resulting from Ca^2+^ cross-linking. The AlgF hydrogel exhibits low strength and Young’s modulus but demonstrates maximum elasticity (Figure 6), which corresponds to a typical hydrated biopolymer network. Moreover, the unfilled AlgF sample shows minimal procoagulant activity and cytocompatibility (see Section 2.3), which can be attributed to its low electron-donor surface energy component γ_s_^−^ that limits polar protein adsorption and initial cell attachment, combined with an insufficient mechanical stiffness required for proper cell spreading.

The introduction of 1 wt% magnetite results in a homogeneous distribution of magnetic nanoparticles within the alginate matrix. Due to their like charges, these nanoparticles partially disrupt the polymer network structure, as evidenced by a decrease in the intensity of the ν(O-H) band in the low-frequency region (<3300 cm^−1^), which corresponds to strong hydrogen bonds (Figure 1), and the disappearance of the thermal effects associated with the glass transition of the alginate matrix (Figure 5). This microstructural alteration limits tight chain packing during drying, leading to an increase in film thickness (Figure 4), a marked decrease in the mechanical properties of the hydrogel (Figure 6), and the emergence of slight magnetic sensitivity (Figure S9).

Increasing the Fe_3_O_4_ content to 5 wt% results in the flocculation of a proportion of the nanoparticles during hydrogel preparation, driven by the screened electrostatic repulsion at elevated ionic strength, which lowers the DLVO energy barrier and promotes bridging flocculation. Subsequent slow drying introduces strong capillary forces and spatial confinement, driving the structural reorganization of these initial isotropic seeds into large elongated MNP clusters aligned along the plane of the film during drying (Figure 4 and Figure S4). These clusters are capable of compacting and reinforcing the alginate matrix. They coexist with particles that are uniformly distributed throughout the sample, contributing to the formation of a continuous, interconnected network morphology acting as a rigid framework. Ultimately, this structure leads to a marked reduction in film thickness back to 48 µm (Figure 4), and a significant enhancement in mechanical strength and stiffness (Figure 6).

At a Fe_3_O_4_ content of 10 wt%, further filler aggregation results in the formation of denser but smaller MNP clusters, predominantly oriented along the plane of the film (Figure 4). Notably, filler agglomerates become clearly visible on the surface of this sample (Figure 3d), whereas for AlgMNP-5 such surface defects were not detected (Figure 3c). As an excessive proportion of nanoparticles participate in coagulation, the continuous percolation framework created by the coexistence of clusters and particle “bridges” likely collapses in this sample. EDX mapping of cross-sections (Figure 4) reveals a distinct spatial inhomogeneity in the iron and carbon distribution, confirming the disruption of uniform dispersion. As a result, the mechanical deterioration at 10 wt% MNPs arises from a combined mechanism. This includes the local disruption of the crosslinked network around the MNP clusters due to the competitive binding of Ca^2+^ ions by citrate groups. Furthermore, under tensile loading, these isolated, rigid, and dense clusters act as internal stress concentrators within the polymer matrix. These effects promote premature crack initiation and propagation, causing a significant decrease in tensile strength and modulus accompanied by a slight preservation of elasticity (Figure 6) and a minor reduction in film thickness (Figure 4).

In the highly filled AlgMNP-20 sample, stratification of the iron-containing phase across the film thickness is observed, characterized by iron enrichment in the lower region and depletion in the upper region (Figure 4). This phenomenon results from the sedimentation of large magnetite-containing clusters during the slow hydrogel formation and drying stages. The resulting asymmetrical filler distribution yields a monolithic material with a well-defined compositional gradient, wherein both layers maintain structural integrity through the continuous, molecularly entangled alginate matrix. The rigid bottom layer, heavily packed with sedimented clusters, serves as a load-bearing reinforcing component, while the upper polymer-rich layer retains high elasticity, promoting crack blunting and stress dissipation at the interlayer interface. This structure contributes to an increase in both strength and Young’s modulus, while maintaining high elasticity without interlayer delamination (Figure 6). Furthermore, the increased concentration of magnetic filler ensures strong interaction with a weak external magnetic field (Figure S9).

The biological performance of the composites correlates positively with the MNP loading (see Section 2.3). The increased electron-donor surface properties (γ_s_^−^ up to 61.9 mJ/m^2^, Figure S8) and the advanced microrelief at higher MNP concentrations (Figure 3c,d) contribute to enhanced procoagulant activity. This correlation is well established for biomaterials: the formation of thrombin and fibrin is initiated by the adsorption and activation of clotting factor XII (Hageman factor) on negatively charged surfaces, triggering the blood clotting cascade [48]. A more complex mechanism underlies the increased adhesion and proliferation of fibroblasts on the film surface, which correlates with higher magnetite content. This mechanism likely involves multiple factors and is associated not only with changes in the donor–acceptor properties of the surface but also with its topography, including the size and density of surface elements [49]. Thus, the improved cytocompatibility at 10 and 20 wt% MNPs is directly governed by the development of a favorable microrelief and increased surface charge density, which cooperatively promote protein adsorption, integrin-mediated cell attachment, and subsequent cell spreading. Importantly, the absence of cytotoxicity at high MNP loadings can be attributed to the effective encapsulation of the nanoparticles within the alginate matrix, which restricts their direct contact with cells and limits the release of potentially toxic iron ions. This is supported by the hemolysis data (Table S4), where the ODS remained at the baseline level even for the AlgMNP-20 sample. However, we acknowledge that these data reflect short-term exposure only. Systematic long-term stability studies are needed to fully assess the safety of these materials in clinical settings.

When magnetite is introduced at a concentration of 20 wt%, a unique synergistic combination of mechanical, magnetic, surface and biological properties emerges, making this material a promising composite for developing wound dressings with procoagulant activity. This includes applications for treating deep and extensive wounds by creating a proregenerative environment that supports the functional activity of fibroblasts. It should be noted that while this pronounced procoagulant activity is highly advantageous for hemostatic wound dressings, it simultaneously poses a significant thrombogenic risk for applications requiring direct blood-contact surfaces, such as vascular grafts or cardiovascular implants.

While the opacity at 10 and 20 wt% MNPs provides effective light shielding, it may limit direct visual inspection of the wound site. This trade-off could be managed by using these materials as temporary protective layers or for short-term applications. Additionally, the exact impact of dense cluster assembly on oxygen diffusion and water vapor transmission rates remains to be quantified. A comprehensive evaluation of these properties represents an essential direction for our forthcoming studies.

## 3. Conclusions

The concentration-driven evolution of the microstructure and functional properties of alginate hydrogels and xerogels containing up to 20 wt% citrate-modified magnetite nanoparticles has been elucidated. At a low loading of 1 wt% MNPs, the individual, similarly charged nanoparticles are uniformly distributed throughout the matrix and incorporated into the alginate interchain spaces, partially disrupting the native network structure and reducing mechanical strength. At 5 wt% Fe_3_O_4_, particle flocculation produces large elongated clusters aligned along the film plane, which form an interconnected reinforcing network that compacts the matrix, thereby substantially enhancing both the mechanical strength and stiffness. At 10 wt% Fe_3_O_4_, the formation of numerous smaller but denser clusters introduces stress concentration sites, leading to a sharp decrease in mechanical properties. In the highly filled sample (20 wt%), stratification into a lower MNP-rich reinforcing layer and an upper polymer-rich elastic layer, which remain interconnected through the continuous alginate matrix, was observed, caused by the sedimentation of large Fe_3_O_4_ clusters during hydrogel formation. This structure provides a synergistic combination of high strength and rigidity while maintaining elasticity without interfacial delamination.

The potential of magnetically sensitive composites for developing cytohemocompatible wound dressings with procoagulant activity has been demonstrated. These materials are suitable for treating deep and extensive wounds by creating a proregenerative environment that supports the functional activity of fibroblasts.

The concentration-dependent microstructural transitions observed in this study enable systematic control over the thermal, optical, and mechanical properties of the composites. This approach opens up opportunities for developing functional hydrogels with tailored properties, including anisotropic ones. The magnetic properties of the filler further suggest the possibility of applying external magnetic fields during the drying stage to spatially orient MNPs within the alginate matrix. This could yield biomaterials with tailored anisotropic mechanical properties, supporting wound healing through directed tissue growth and replicating native tissue architecture.

Future work should include quantitative magnetization measurements (VSM) and focus on utilizing external magnetic fields to manipulate the microstructure and induce anisotropic particle alignment during processing. Subsequent studies should also involve the evaluation of magnetically induced deformation and drug release, gas permeability, water vapor transmission rates, and long-term stability assessment (including iron ion leaching and matrix degradation). In vivo validation will also be essential for clinical translation.

## 4. Materials and Methods

### 4.1. Materials

Na-Alginate (Alfa Aesar, Haverhill, MA, USA; low viscosity, M_w_ = 4.1 × 10^5^ by gel permeation chromatography; M/G ratio of 1.67 ± 0.17, determined using NMR spectroscopy [50]), CaCl_2_∙6H_2_O (Reakor, Moscow, Russia, >98%,), FeCl_2_·4H_2_O (Sigma-Aldrich, St. Louis, MO, USA, >99.0%), FeCl_3_·6H_2_O (Sigma-Aldrich, 97.0–102.0%), anhydrous citric acid (RusChim, Moscow, Russia, 99%), ammonia (ChimMed, Moscow, Russia, >99.9%), and deionized water were used in the experiments, along with Calcein AM Cell Viability Assay Kit (Servicebio, Wuhan, China), FITC Annexin V Apoptosis Detection Kit (Pharmingen, San Diego, CA, USA), a solution of 0.02 M CaCl_2_ (set R-9/1 NPO Renam, Moscow, Russia), a set of reagents for determining activated partial thromboplastin time (APTT) (PG-7/1 NPO Renam, Moscow, Russia), and phosphate-salt buffer (PBS), tableted, 0.01 M, pH 7.4 (Amresco, Solon, OH, USA).

### 4.2. Methods

#### 4.2.1. Preparation of Magnetite Nanoparticles

A negatively charged citrate-modified magnetite sol was synthesized by co-precipitation of FeCl_2_·4H_2_O (Sigma-Aldrich, >99.0%) and FeCl_3_·6H_2_O (Sigma-Aldrich, 97.0–102.0%) in an alkaline medium, followed by surface functionalization with citric acid and ultrasonication, as described in detail elsewhere [27].

#### 4.2.2. Preparation of Hydrogels

A 2 wt% sodium alginate (Na-Alg) solution was prepared by dissolving the polymer in water at 70 °C with stirring for 4 h. To prepare composite films, the initial water volume was adjusted for the later MNP hydrosol addition. This kept the final Na-Alg concentration at 2 wt% for all samples. The calculated volume of the MNP hydrosol (1, 5, 10, or 20 wt% Fe_3_O_4_ relative to dry mass of Na-Alg) was then added with rapid stirring at 70 °C. The resulting solutions were left stirred for 60 min while cooling to 40 °C, after which they were poured directly into polystyrene Petri dishes. The volume of solution in each dish was controlled with an electronic scale, so the dry Na-Alg mass per 10 cm^2^ was 52 mg. The Na-Alg/MNP gels were dried at 40 ± 1 °C (5 ± 2% humidity), cross-linked in 8 wt% CaCl_2_ for 60 min (for impart water insolubility), washed with water to remove excess CaCl_2_ (conductometric monitoring), and re-dried on a polystyrene surface at 40 ± 1 °C (4 ± 1% humidity). All processing parameters, including stirring duration, drying temperature, and ionotropic gelation time, were kept strictly constant across all samples to ensure reproducible kinetics of cluster formation and enable a systematic comparison across different compositions. The resulting films were designated AlgMNP-X, where X is the represents Fe_3_O_4_ mass content percentage (wt%) relative to dry mass of alginate (e.g., AlgMNP-10). Alginate films without added nanoparticles were designated AlgF.

#### 4.2.3. Methods of Studying Initial Nanoparticles and Polysaccharide Films

Detailed experimental procedures for Fourier-transform infrared (FTIR) and ultraviolet–visible (UV-Vis) spectroscopy, zeta potential measurements, X-ray diffraction (XRD), transmission and scanning electron microscopy (TEM and SEM), optical microscopy, synchronous thermal analysis, and contact angle measurements are provided in the Supporting Information. The methods for assessing the mechanical properties, magnetic sensitivity, biocompatibility, and hemocompatibility of the hydrogels are also described therein.

## Data Availability

Data are contained within the article and available upon request.

## Supplementary Materials

The following supporting information can be downloaded at: https://www.mdpi.com/article/10.3390/gels12090786/s1, Materials and methods: Table S1. Values of free surface energy components for test liquids used in this work. Figure S1. XRD (a) and TEM micrographs (b) of MNPs. Figure S2. Digital photographs of AlgMNP films depending on the mass fraction of magnetite. Figure S3. UV–visible transmission spectra of AlgF and AlgMNP films normalized to a reference thickness of 50 µm. Figure S4. Optical micrographs of AlgMNP-1 (a–e) and AlgMNP-5 (b–f) films obtained on Petri dishes at different magnifications in reflection mode on a stereomicroscope. Figure S5. SEM micrographs (a–c,e) in SE, BSE, and EDX mapping modes (d,f) of AlgMNP-20 film. Figure S6. Box chart of cluster length and thickness in AlgMNP-5 and AlgMNP-10 composite films determined from cross-sectional SEM image analysis (*n* > 10 clusters per sample). Asterisks denote statistically significant differences between the two samples for both length and thickness (Mann–Whitney U-test, *** *p* < 0.001). Figure S7. SEM micrograph of AlgF film cross-section. Figure S8. Dependences of γ_s_^LW^ γ_s_^+^, and γ_s_^−^ on the mass fraction of magnetite in the alginate matrix. Figure S9. Visual demonstration of the macroscopic magnetic responsiveness of the AlgMNP composite films: AlgMNP-5 (a–e), AlgMNP-10 (f–j) and AlgMNP-20 (k–o). The photographs show the interaction distance between the film samples and a permanent NdFeB magnet (class N52). Figure S10. Length of human fibroblasts after 24 h. Data are expressed as Mean ± SD; *—differences are significant compared to control, at *p* < 0.05, *n* = 5. Table S2. Color parameters (L*, a*, b*) of the prepared composite films. Table S3. Contact angle values of various test liquids for AlgF and AlgMNP compositions. Table S4. Optical density (545 nm) of the supernatant fluid (ODS) obtained by centrifugation of a suspension of erythrocytes pre-incubated with AlgMNP composite films with an area of (S_1_) 0.25 cm^2^ and (S_2_) 0.56 cm^2^. References [44,45,46,51,52,53,54,55] are cited in the Supplementary Materials file.
