# Supplementary material for: Magnetite Concentration-Driven Evolution of Microstructure and Property Tuning in Alginate Hydrogels
Source: Gels. 2026 Sep 1;12(9):786. doi: 10.3390/gels12090786 (PMC13606123; doi:10.3390/gels12090786)
Supplement: Supplementary file 1 [file gels-12-00786-s001.zip › gels-4447175-supplementary.pdf]

Supporting Information

Magnetite concentration-driven evolution of microstructure and property tuning in alginate hydrogels

Vasily I. Mikhaylov[1,*], Mikhail A. Torlopov[1], Ilia S. Martakov[1,*], Philipp V. Legki[1], Kirill S. Vavrinchuk[1], Pavel A. Markov[2], Natalia N. Drozd[3], Kirill A. Cherednichenko[4], Petr A. Sitnikov[1]

* Correspondence: gmartakov@gmail.com (I.S.M), system14@rambler.ru (V.I.M.)

Table of Contents

**1. Materials and methods**	**S1**
**1.1 Characterization of Nanoparticles and Films**	**S1**
**1.2. Biological and Hemocompatibility Assays**	**S4**
**2. Results**	**S7**
**2.1 Supplementary Figures**	**S7**
**2.2 Supplementary Tables**	**S13**
**3. References**	**S13**

1. Materials and methods

1.1 Characterization of Nanoparticles and Films

The FTIR spectra of dried MNPs and films were recorded using a Prestige 21 FTIR spectrometer (Shimadzu, Japan) with an interval of 2 cm^{-1} in the region of 4000–400 cm^{-1} in diffuse reflectance mode.

Gel permeation chromatography. The chromatographic system employed was a Shimadzu (Japan) setup, which included an LC-20 AD pump, a STO-10 AS thermostat, a RID-10 A refractometer, and a Shodex OHpac SB804 HG column (8 mm × 30 cm). Elution was conducted with a 0.15 M NaCl solution at 40°C, with a flow rate of 0.3 cm^3/min. To calibrate the column, samples of sulfated pullulan with molecular weights ranging from 1.3×10^3 to 8×10^3 were utilized.

The UV-visible spectra of the dried films were recorded using a Solar PB 2201 spectrophotometer in the wavelength range of 200–800 nm with a step of 0.5 nm in transmission mode. To eliminate the effect

of film thickness variation on the optical transmittance, the UV–Vis spectra were normalized to a reference thickness ($l_{ref.}$) of 50 μm using the relation $T_{\text{norm.}} = T^{l_{\text{ref}}/l}$, where T is the measured transmittance and l is the actual film thickness (in μm). This normalization assumes that light attenuation is dominated by absorption; however, some contribution from scattering cannot be excluded.

The mean hydrodynamic diameter, polydispersity index, and zeta potential of MNPs in sols were determined by dynamic light scattering and laser Doppler electrophoresis (Malvern ZetaSizer Nano ZS, 4 mW He/Ne laser, 633 nm), at 25 °C in a DTS1070 disposable capillary cell. All the measurements were taken at least three times.

XRD patterns of freeze-dried MNPs were recorded by an XRD-6000 diffractometer (Shimadzu) using CuKα radiation (λ = 1.541 Å).

Transmission electron microscopy (TEM) images of MNPs were recorded on an electron microscope JEM-1400 (Jeol, Japan), at 80 kV. Copper grids coated by carbon were used for imaging dry particles.

Optical micrographs (OM) of the film surfaces were obtained using an Olympus BX53M microscope equipped with a 50x objective and a 20 MP camera. Xerogels for optical microscopy were prepared from standard Na-Alg solutions with a specified amount of Fe_3O_4 sol added. A thin layer of the solution was applied to a glass slide, dried in a convection oven at 40 °C, and then immersed in an 8 wt% aqueous $CaCl_2$ solution for 10 minutes. The sample was then rinsed in water to remove excess salts and dried for 2 hours at 40 °C.

The surface and cross-sectional morphology of dried alginate films and their derivatives modified with magnetite nanoparticles was examined using a Vega3 SBU scanning electron microscope (Tescan, Czech Republic) in backscattered electron (BSE) and secondary electron (SE) modes. Prior to examination, the sample was coated with a 3 nm thick conductive gold layer using a DSCR magnetron sputtering system (Nanostructured Coatings Co., Iran). EDX mapping of the magnetite-containing films was performed using an AZtec Energy/X-act energy-dispersive X-ray microanalyzer (Oxford Instruments, UK) coupled to a Vega3 SBU scanning electron microscope.

Quantitative analysis of cluster dimensions for AlgMNP-5 and AlgMNP-10 was performed on SEM images using Gwyddion software (v. 2.69). Statistical significance was assessed using the Mann–Whitney U-test (p < 0.001). Data are presented as box-and-whisker plots representing the median, mean, and 1.5×interquartile range (IQR) whiskers.

Simultaneous thermal analysis (STA) of the dried films was performed using a NETZSCH STA 449F5 instrument in aluminum crucibles. Sample weights were 15–20 mg, the temperature range was 25–300 °C, the heating rate was 10 °C/min, and the nitrogen purge rate was 50 cm^3/min. The glass transition temperature (T_g), melting temperature (T_m), decomposition temperature (T_d), and enthalpy of fusion (ΔH_m) of the samples were obtained from DSC thermograms. The thermal analysis was limited to 300 °C because all key transitions relevant to the polymer network and filler–matrix interface are completed below this threshold. Heating beyond this temperature would only provide non-specific mass loss from carbonaceous residue degradation.

The tensile mechanical properties of hydrogels pre-swollen in Hanks' solution at pH 5.5 were evaluated under tensile loading using ST-2 texture analyzer (Quality Laboratory, Russia). Film samples measuring 70 x 8 mm were clamped vertically in a steel vice at a distance of 30 mm. The samples were then ruptured at a rate of 1 mm/s. The initial load was 10 g. This resulted in stress–strain curves in g/s coordinates. All tests were performed at least five times. Young's modulus (MPa) was subsequently calculated using the formula:

$$E = \frac{S_0 * L_0}{A_R}, (1)$$

where S_0 is the slope of the linear region on the stress–strain curves, L_0 is the distance between the clamps, and A_R is the cross-sectional area of the specimen.

The tensile strength was determined using the formula:

$$\sigma = \frac{Fmax}{A_R}, (2)$$

where F_{max} is the maximum stress.

Elongation (%) was calculated using the formula:

$$\varepsilon = \frac{L_{max}}{L_0} * 100, (3)$$

where L_{max} is the length at break.

Statistical analysis of mechanical properties was performed using OriginPro 2024. The Kruskal–Wallis test was used to assess overall differences between groups. Post hoc pairwise comparisons were conducted using Conover's test. The significance level was set at $p < 0.05$. Data are presented as mean ± standard deviation (SD) from at least five independent measurements ($n \geq 5$). Different letters above bars indicate statistically significant differences between groups, with the letter «a» assigned to the highest mean value. Groups sharing the same letter are not significantly different.

The contact angle of the test liquids was determined using the SEO Phoenix MT (M.A.T.) instrument complex using the sessile drop method. Samples for contact angle measurements were prepared on glass substrates in a manner similar to that used for optical microscopy. The surface free energy components of composites containing varying ratios of Fe_3O_4 and alginate were determined using the extended Young equation (van Oss–Good–Chaudhuri approach) (4), which takes into account both nonpolar and polar interactions [1,2]:

$$(1 + \cos\theta) \cdot \gamma_l^{TOT} = 2 \cdot \left(\sqrt{\gamma_s^{LW} \cdot \gamma_l^{LW}} + \sqrt{\gamma_s^{+} \cdot \gamma_l^{-}} + \sqrt{\gamma_s^{-} \cdot \gamma_l^{+}} \right) (4)$$

where θ is the contact angle of a liquid droplet on a solid surface, the subscripts *l* and *s* refer to the liquid and solid, respectively, γ_l^{TOT} is the total free surface energy of the liquid, γ^{LW} are the nonpolar (dispersive) components of the free surface energy associated with the Lifshitz–van der Waals forces, and γ^+ and γ^- are the free surface energy components of electron acceptors (acid component) and donors (basic component), respectively.

To determine the nonpolar (dispersive) component of the free surface energy of the substrate γ_s^{LW}, a nonpolar liquid (diiodomethane) was used, for which $\gamma_l^+ = \gamma_l^- = 0$ and $\gamma_l^{LW} = \gamma_l^{TOT}$. In this case, the last two terms of the right-hand side of Equation (4) become equal to zero, and the equation is simplified:

$$\gamma_s^{LW} = \frac{\gamma_l^{TOT} \cdot (1+\cos\theta)^2}{4}. \quad (5)$$

The values of the acidic γ_s^+ and basic γ_s^- components of the free surface energy of the substrate were determined by solving a system of two equations with two unknowns (6), compiled for two polar test liquids:

$$\begin{cases} (1+\cos\theta_{H_2O}) \cdot \gamma_{H_2O}^{TOT} = 2 \cdot \left(\sqrt{\gamma_s^{LW} \cdot \gamma_{H_2O}^{LW}} + \sqrt{\gamma_s^+ \cdot \gamma_{H_2O}^-} + \sqrt{\gamma_s^- \cdot \gamma_{H_2O}^+}\right) \\ (1+\cos\theta_l) \cdot \gamma_l^{TOT} = 2 \cdot \left(\sqrt{\gamma_s^{LW} \cdot \gamma_l^{LW}} + \sqrt{\gamma_s^+ \cdot \gamma_l^-} + \sqrt{\gamma_s^- \cdot \gamma_l^+}\right) \end{cases} \quad (6)$$

where the subscripts *l* and H_2O refer to the polar test liquid and water, respectively.

To improve the accuracy of the determination, data were used for several polar liquids (water, formamide, glycerol, and ethylene glycol), sequentially solving systems where known (free surface energy components) and experimentally obtained (wetting angle) values were substituted into the second equation. The solutions of the corresponding systems of equations were averaged to avoid large discrepancies between simultaneous solutions of the equations [3].

The values of the surface free energy components of the liquids used were taken from the works [3,4] and are presented in Table S1.

Table S1. Values of free surface energy components for test liquids used in this work

Test liquid	γ_l^{TOT}, mJ/m^2	γ_l^{LW}, mJ/m^2	γ_l^+, mJ/m^2	γ_l^-, mJ/m^2
Water	71.8	21.8	25.5	25.5
Formamide	57.9	39.0	2.3	39.6
Glycerol	64.0	34.0	3.92	57.4
Ethylene glycol	48.0	29.0	1.92	47
Diiodomethane	50.8	50.8	0	0

Samples for contact angle measurements were prepared on glass substrates in a manner similar to those prepared for optical microscopy.

To assess the *magnetic sensitivity* of the hydrogels, an experiment was conducted to measure their attraction to a permanent neodymium magnet (class N52, 50 mm in diameter, 30 mm thick). For this experiment, pre-swollen films were fixed to a stand clamp in a suspended (vertical) position at a specified distance from a permanent magnet mounted parallel to the film. The stand clamp with the film was then gradually moved toward the magnet while photographing the samples at various distances. A ruler was used to record the initial distance of film–magnet interaction, when the sample first begins to move toward the magnet and deviates from the vertical position (trigger distance, H_T).

1.2. Biological and Hemocompatibility Assays

Biological assay

Fibroblast adhesion. The adhesive properties of the biomaterial were assessed based on the number of fibroblasts attached to the sample surface. Samples, pre-sterilized with alcohol and UV irradiation, were placed into the wells of 96-well plates, one sample per well, with a total of 7 replicates for each sample.

DMEM-F12 culture medium containing 10 % FBS and 1 % penicillin-streptomycin solution was used in all experiments unless otherwise stated. Then, 0.1 ml of cell suspension containing $3.5\text{-}4.0 \times 10^4$ fibroblasts/ml was added to the wells. The samples were incubated under standard conditions (37 °C, 5 % CO_2). After 6 h, the samples were removed, washed with PBS to remove non-adherent cells, and then fixed with 70 % ethyl alcohol solution (-20 °C). After drying at room temperature, the samples were successively coated with DAPI and Rhodamine fluorescent dyes. After 10 minutes, excess dye was removed with PBS. The samples were placed on glass slides and fixed with coverslips. Fibroblast numbers and morphometric characteristics were assessed using light and fluorescence microscopy. Cell adhesion was assessed by counting cells in 20 squares of 200 μm^2 using Leica Application Suite software (Leica Microsystems).

Morphometric characterization of cells and assessment of fibroblast proliferative activity. Proliferative activity was assessed by co-incubation of the studied samples with human fibroblasts for 48 h. A cell suspension (1 ml) containing fibroblasts (5×10^4 pcs/ml) was added to the wells of a 12-well culture plate and incubated for 60 minutes under standard conditions to ensure cell attachment to the well surface. Then, samples (ø10 mm), pre-sterilized with alcohol and UV irradiation, were placed into the wells of the plates at a rate of one sample per well, with a total of 7 replicates for each sample. Incubation was carried out under standard conditions (37 °C, 5 % CO_2). The number and morphometric characteristics of fibroblasts were assessed after staining with DAPI and Rhodamine fluorescent dyes after 24 and 48 h of incubation. Proliferative activity was assessed by counting the number of cells in 20 squares of 200 μm^2 using Leica Application Suite software (Leica Microsystems).

Evaluation of metabolic activity of fibroblasts. Metabolic activity was assessed by co-incubation of the studied samples with human fibroblasts for 24 h. The cell suspension (0.1 ml) containing fibroblasts ($3.5\text{-}4.0 \times 10^4$ pcs/ml) was added to the wells of a 96-well culture plate and incubated for 60 minutes under standard conditions to allow the cells to attach to the well surface. Then, samples (ø5 mm), pre-sterilized with alcohol and UV irradiation, were placed into the wells of the plates at a rate of one sample per well; the total number of replicates for each sample was 7 pcs. Incubation was carried out under standard conditions (37 °C, 5 % CO_2). After 24 hours, the samples were removed, washed with PBS to remove debris, and then 0.1 ml of DMEM-F12 was added. The MTT viability assay was performed with slight modifications as previously described [5]. MTT was first prepared as a stock solution of 5 mg/mL in phosphate-buffered saline (PBS, pH 7.2) and filtered. At the end of the treatment period (48 h), 20 µL of MTT solution was added to each well. After incubation for 4 h at 37 °C, 100 µL of solubilizing buffer (10 % sodium dodecyl sulfate dissolved in 0.01M HCl) was added to each well. After overnight incubation, the 96-well plate was read by an enzyme-linked immunosorbent assay (ELISA) reader at 570 nm for absorbance density values to determine the cell viability. The viable cells produced a dark blue formazan product, whereas no such staining was formed in the dead cells. The percentage of the viable cells was calculated using the following formula: (%) = [100 × (sample abs)/ (control abs)].

The biological evaluation included the unfilled alginate film (AlgF) as a control, and focused on AlgMNP-10 and AlgMNP-20, as these compositions exhibit surface-exposed magnetite aggregates essential for mediating cell–material interactions. AlgMNP-1 was excluded due to its poor mechanical

properties, which make it unsuitable for biomedical applications. AlgMNP-5 was excluded because no magnetite aggregates are present on its surface, and its biological performance was therefore expected to be similar to that of AlgF.

Analysis of the effects of hydrogel materials and their components on blood and plasma coagulation in vitro

Hemocompatibility. AlgF and AlgMNP films were cut into squares measuring 0.5 cm ×0.5 cm (S_1 = 0.25 cm^2) and 0.75 cm×0.75 cm (S_2 = 0.56 cm^2) using templates. Prior to the experiments, the films were incubated in 0.5 ml of 0.9 % NaCl solution (saline solution) at room temperature. Human blood samples were stabilized with 3.2 % trisodium citrate ($Na_3C_6H_6O_7$) (vacuum tubes “UNIVAC" 13100.5.4.TSN3.2. for venous blood collection; LLC “Eiliton” by order of CJSC “Unimed”, Russia), obtained from the elbow veins of donors of both sexes. Blood was received from 23.12.2024 to 30.01.2025. All donors signed the "Informed Voluntary Consent for Medical Examination and Donation of Blood and (or) Its Components" (Appendix No. 2 to the Order of the National Medical Research Center of Hematology of the Ministry of Health of the Russian Federation dated August 2, 2023, No. 475). The study protocol was approved at a meeting of the problem commission "Fundamental and Clinical Research in Hematology; Problems of Clinical and Industrial Transfusiology" of the Federal State Budgetary Institution "National Medical Research Center of Hematology" of the Ministry of Health of Russia No. № 185 dated February 27, 2025. Platelet-depleted plasma was obtained by centrifuging the blood for 20 minutes at 3000 rpm using an SM-6M centrifuge (Elmi, Latvia). Experiments were performed as described in the standard operating procedures (N◦LFS, VNAG-A-001/01; N◦LFS, VNAG-A-002/01; N◦LFS, VNAG-A-003/01 dated 01.03.2024) of the Laboratory of Pharmacology of Agents Affecting Hemostasis of National Medical Research Center for Hematology.

Blood recalcification time (BRT) test. A total of 0.5 ml of blood was added to 1.5 ml plastic tubes with films and incubated at 37 °C in the wells of a solid-state thermostat (Thermo Block TDB-120, Biosan, Latvia). To set up the BRT test [6], after 10 or 20 minutes of incubation, 0.05 ml of blood from the test tubes was added to a microcell of a programmable semi-automatic coagulometer (APG2-01 or APG4-03-P, EMCO LLC, Moscow, Russia), and the microcell was placed into the thermostatically controlled well (37 °C) of the device. After 1 minute, 0.05 ml of 0.02 M $CaCl_2$ (MP Biomedicals, USA) was added, and the blood coagulation time was recorded.

Activated partial thromboplastin time (APTT). A total of 0.280 ml of platelet-poor plasma was added to 1.5 ml plastic tubes with films and incubated at 37 °C in the wells of a solid-state thermostat (Thermo Block TDB-120, Biosan, Latvia). To perform the APTT test [7] reagents and instructions from the PG-7/1 kits (RPA Renam, Moscow, Russia) were utilized. After incubation periods of 1 minute, 5 minutes, or 10 minutes, 0.03 ml of plasma and 0.03 ml of APTT reagent were added to the microcell of a programmable semi-automatic coagulometer APG2-01 (EMCO LLC, Moscow, Russia). The microcell was then placed in a thermostatically controlled well (37 °C) of the device. After 3 minutes, 0.05 ml of 0.025 M $CaCl_2$ was added, and the plasma coagulation time was recorded.

Hemolysis of erythrocytes. The effect of films on erythrocyte hemolysis was assessed using the method described in [8]. Blood samples were centrifuged at 250 g for 15 minutes, and the erythrocytes were washed three times with PBS. A tube containing a film, 0.25 ml of PBS, and 0.25 ml of erythrocyte suspension was placed in a solid-state thermostat (Thermo Block TDB-120; Biosan) at 37 °C. After 2 hours, the tubes were centrifuged again at 250 g for 15 minutes. The optical density (OD) of the supernatant obtained after centrifugation was measured at a wavelength of 540 nm using a Smart Spec Plus spectrophotometer (Bio-Rad). The degree of erythrocyte hemolysis (DEH, %) was calculated using the following formula:

$$DEH = (OD_0 - OD_{(K-)}/OD_{(K+)} - OD_{(K-)}) \times 100\ \%, \quad (7)$$

where OD_0 is OD of the supernatant after incubation with samples; $OD_{(K-)}$ is OD of the supernatant after incubation with saline solution; and $OD_{(K+)}$ is OD of the supernatant after incubation with distilled water.

Statistical processing of the obtained results was performed using Primer of Biostatistics 4.03 (The Mc Graw-Hill Companies, Inc.) and Statistica 8.0 (StatSoft, Inc.) software. Non-parametric Mann–Whitney U-test (for unrelated samples) and Wilcoxon t-test (for related samples) were used to compare non-normally distributed data. The study results are presented as arithmetic means ± standard errors of the arithmetic means for determinations of n = 5 or > 5. Differences between data series were considered statistically significant at $p < 0.05$.

2. Results

2.1 Supplementary Figures

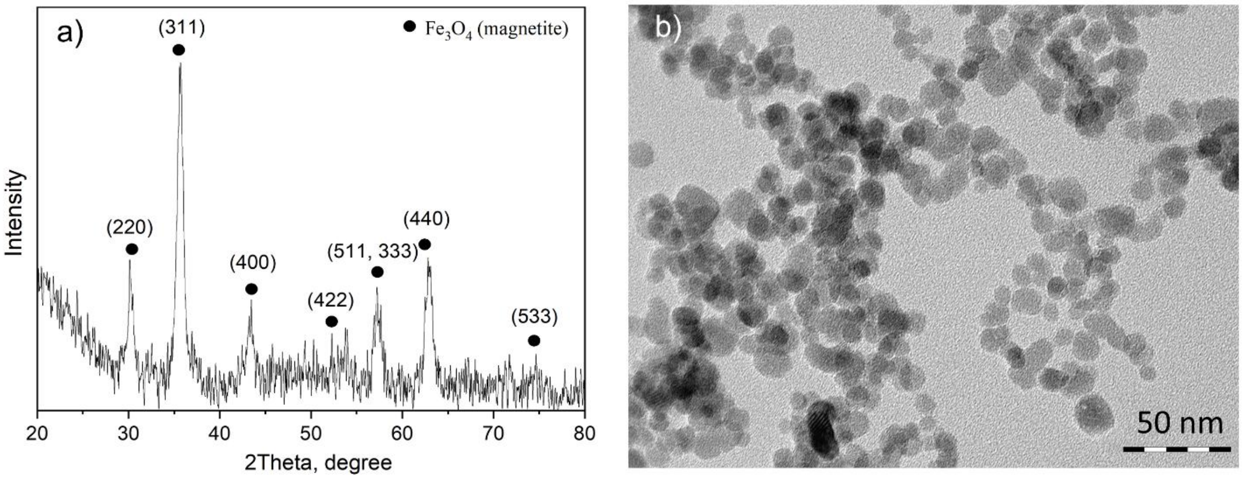

Figure S1. XRD (a) and TEM micrographs (b) of MNPs.

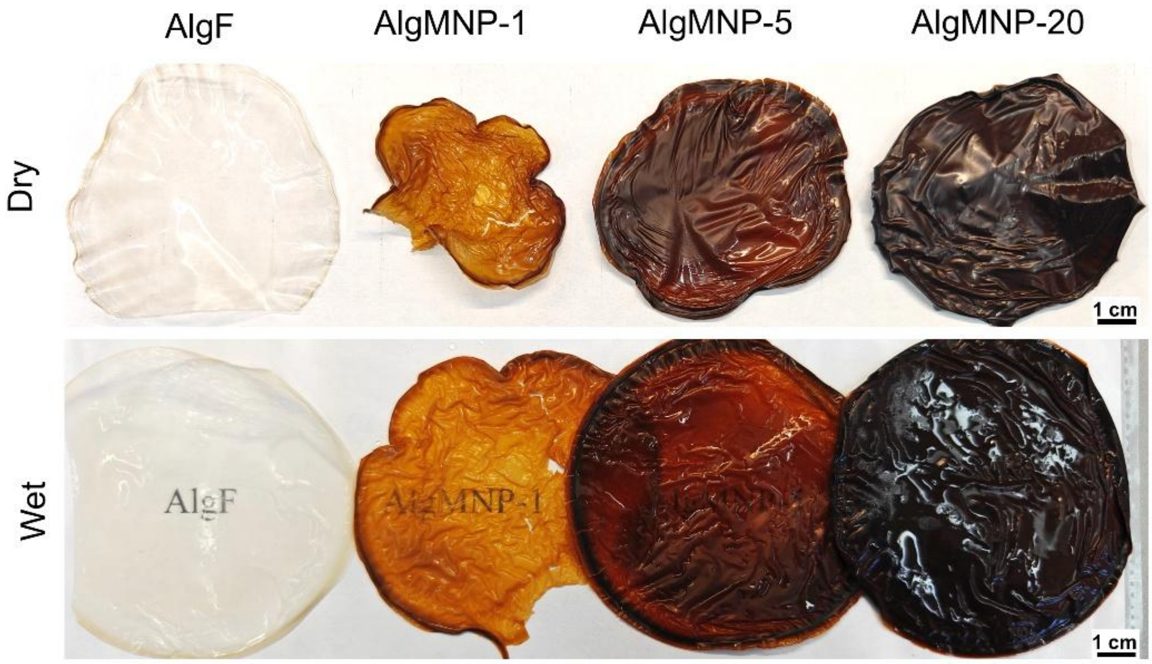

Figure S2. Digital photographs of AlgMNP films depending on the mass fraction of magnetite.

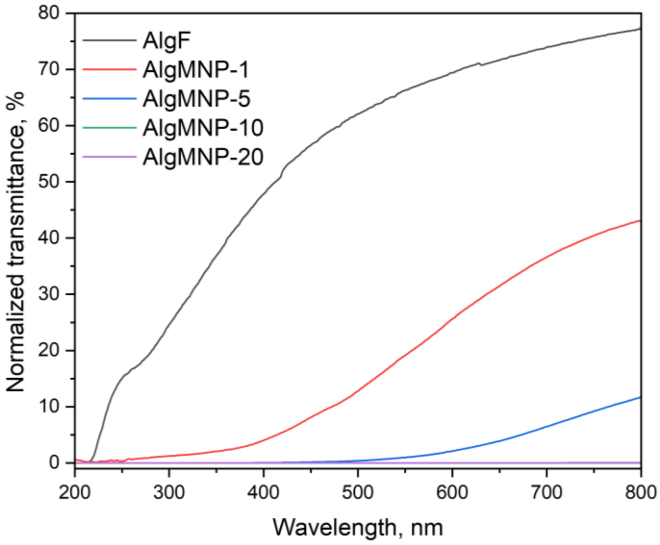

Figure S3. UV–visible transmission spectra of AlgF and AlgMNP films normalized to a reference thickness of 50 μm.

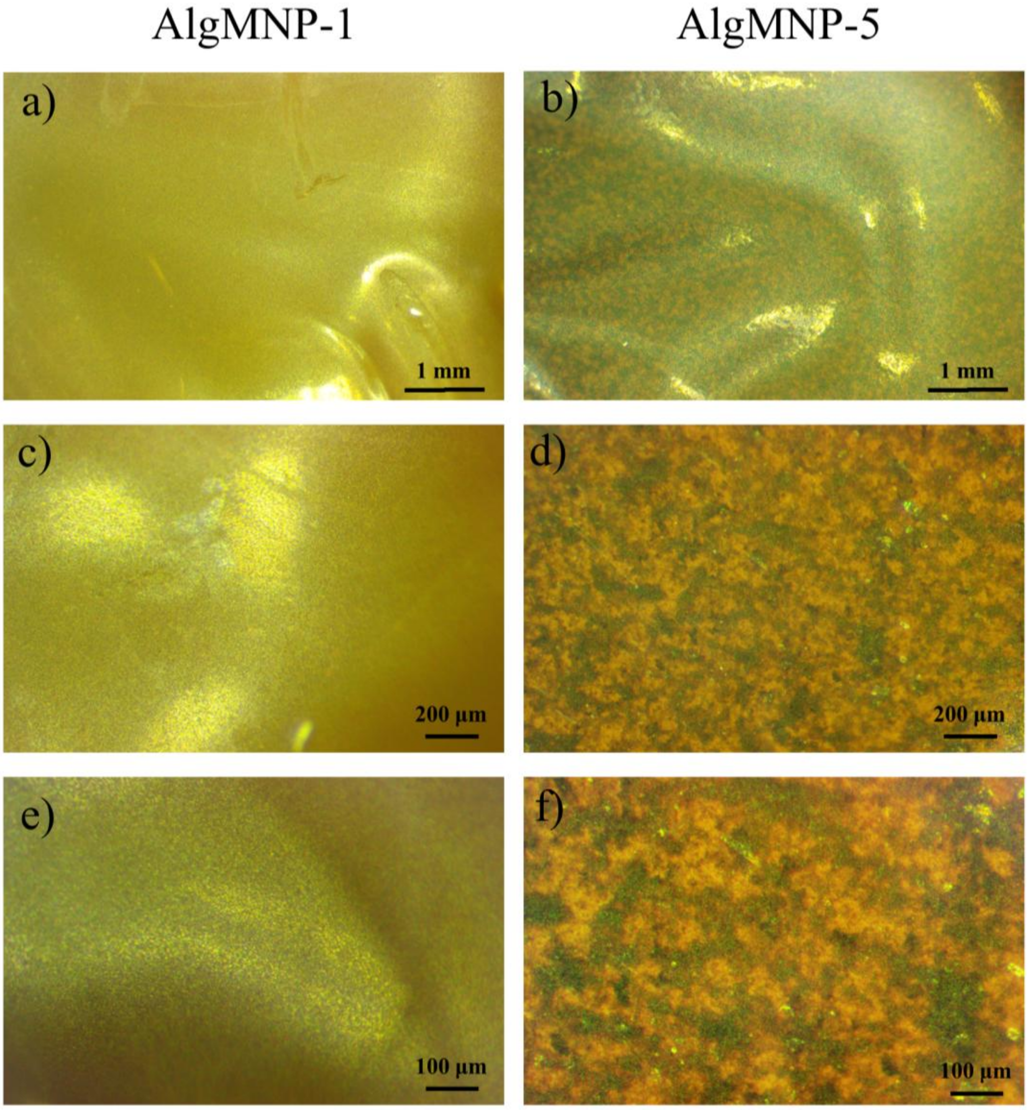

Figure S4. Optical micrographs of AlgMNP films obtained on Petri dishes at different magnifications in reflection mode on a stereomicroscope.

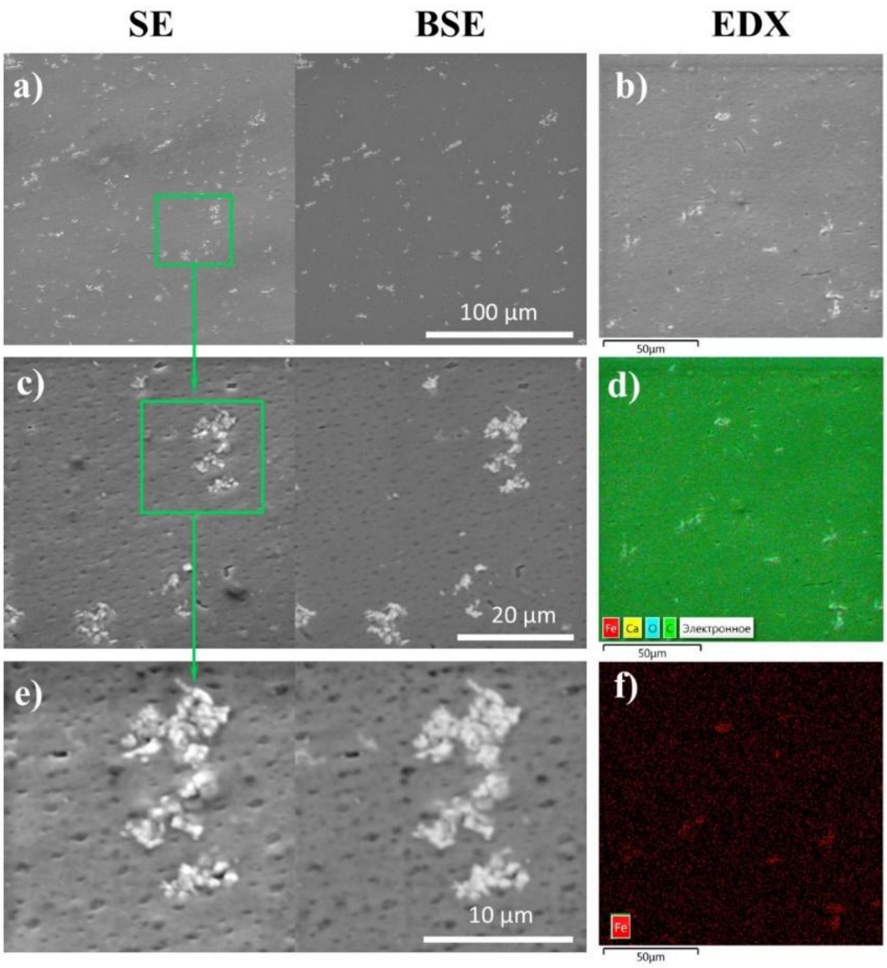

Figure S5. SEM micrographs (a-c, e) in SE, BSE, and EDX mapping modes (d, f) of AlgMNP-20 film.

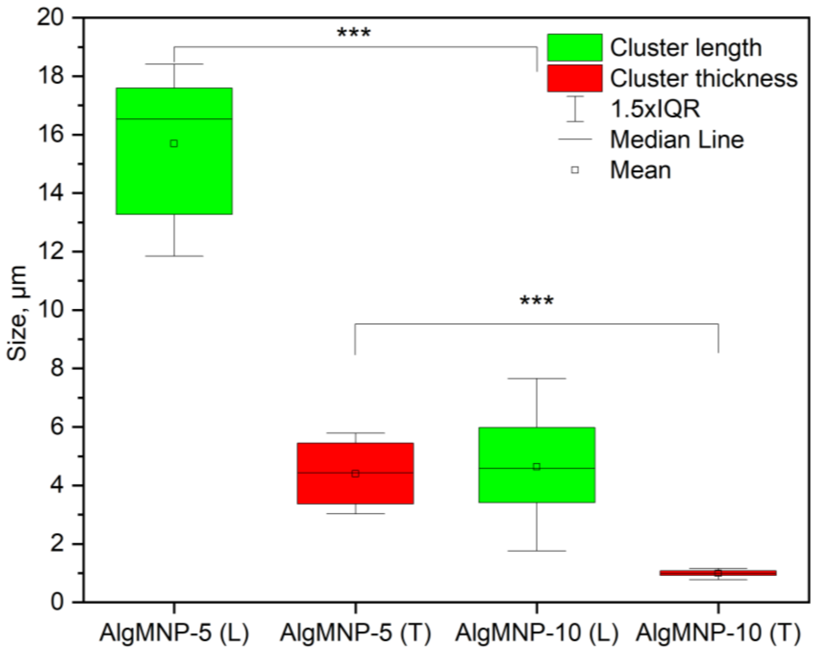

Figure S6. Box chart of cluster length and thickness in AlgMNP-5 and AlgMNP-10 composite films determined from cross-sectional SEM image analysis (n > 10 clusters per sample). Asterisks denote statistically significant differences between the two samples for both length and thickness (Mann–Whitney U-test, *** $p < 0.001$).

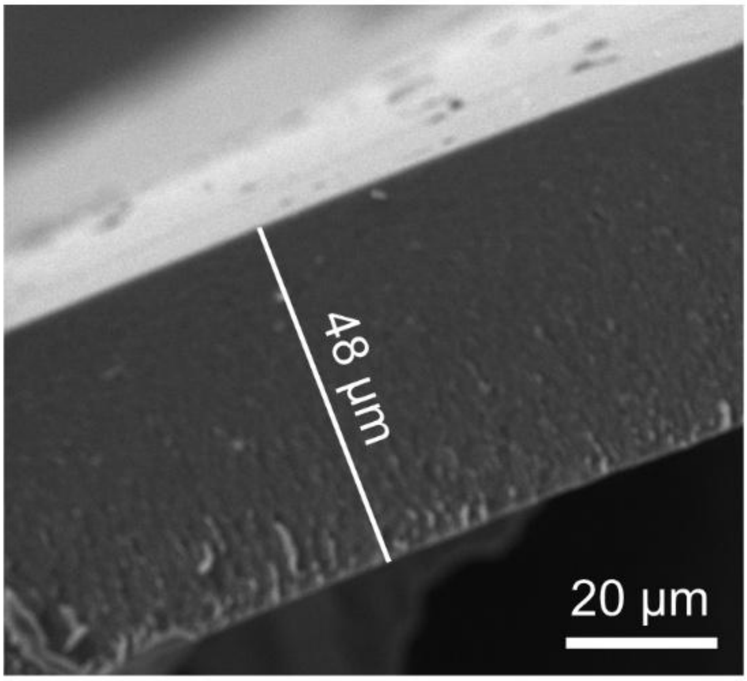

Figure S7. SEM micrograph of AlgF film cross-section.

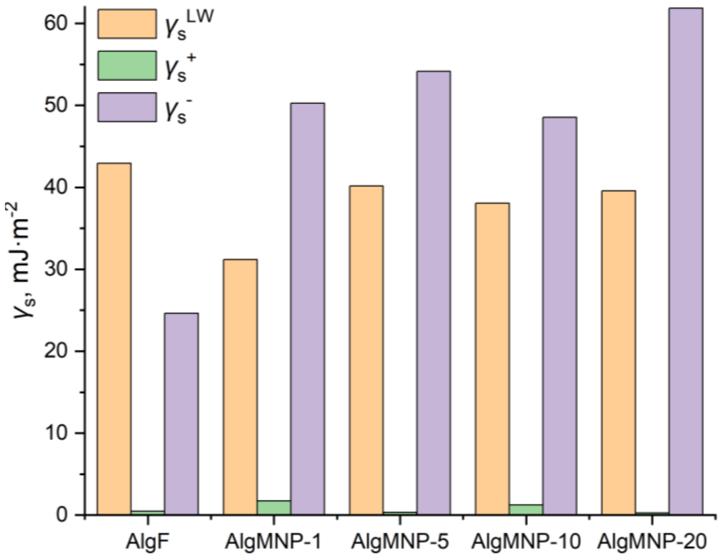

Figure S8. Dependences of γ_s^{LW}, γ_s^{+}, and γ_s^{-} on the mass fraction of magnetite in the alginate matrix.

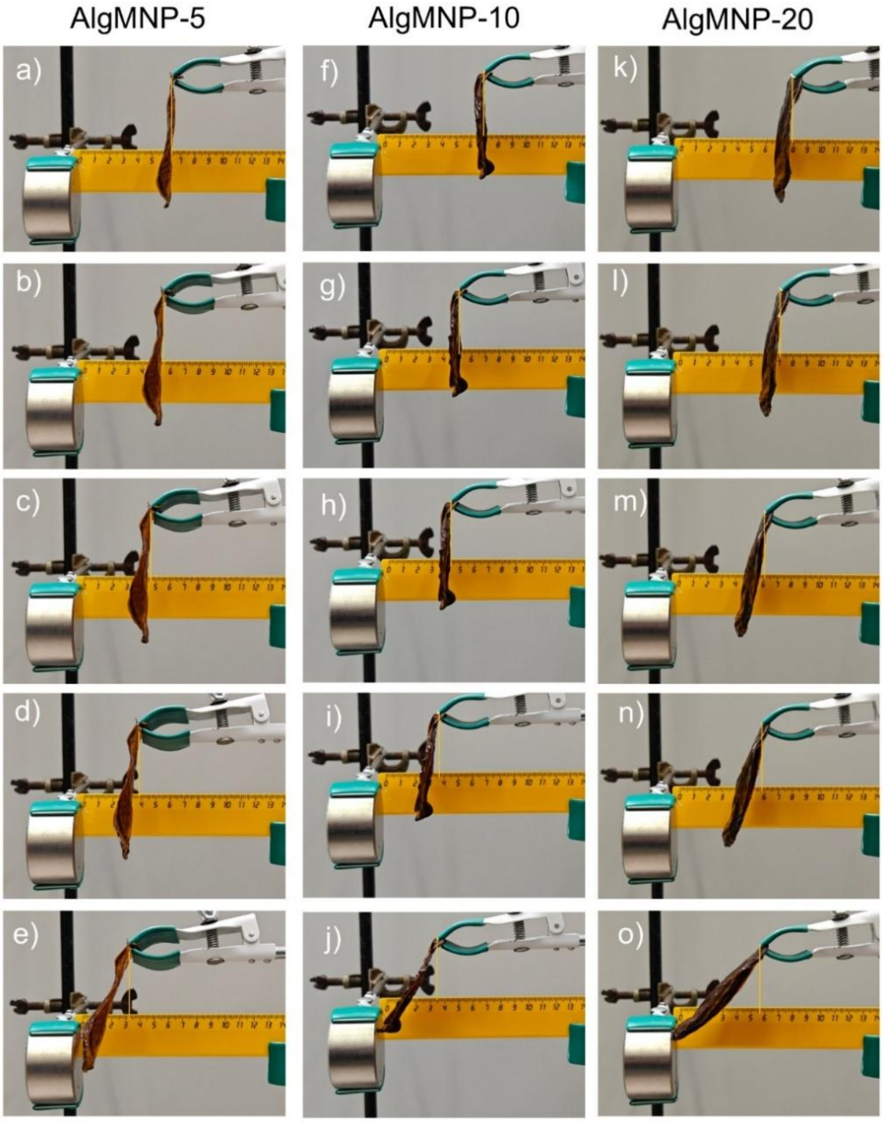

Figure S9. Visual demonstration of the macroscopic magnetic responsiveness of the AlgMNP composite films: AlgMNP-5 (a-e), AlgMNP-10 (f-j) and AlgMNP-20 (k, o). The photographs show the interaction distance between the film samples and a permanent NdFeB magnet (class N52).

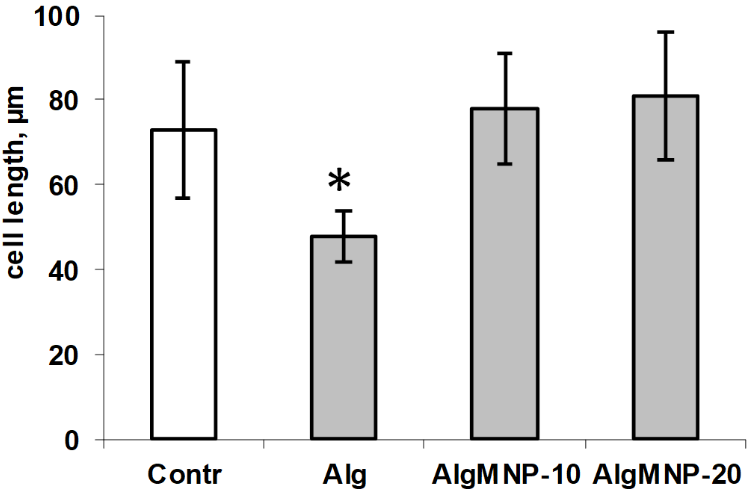

Figure S10. Length of human fibroblasts after 24 hours. Data are expressed as Mean ± SD; * – differences are significant compared to control, at $p<0.05$, n=5.

2.2 Supplementary Tables

Table S2. Color parameters (L*, a*, b*) of the prepared composite films.

Sample	L*	a*	b*	Color
AlgF	85.6	0.95	8.79	Light brown
AlgMNP-1	33.5	12.23	32.30	Brown
AlgMNP-5	12.7	12.45	18.71	Dark brown
AlgMNP-10	0.08	0.26	0.08	Black
AlgMNP-20	0.03	$3.6 \cdot 10^{-4}$	$-1.3 \cdot 10^{-3}$	Black

Table S3. Contact angle values of various test liquids for AlgF and AlgMNP compositions.

Sample	Diiodo-methane	Water	Glycerol	Ethylene glycol	Formamide	γ_s^{LW}, mJ/m^2	γ_s^{+}, mJ/m^2	γ_s^{-}, mJ/m^2
AlgF	33.5±0.2°	51.9±0.4°	49.6±0.3°	36.1±0.4°	36.8±0.5°	42.94	0.45	24.64
AlgMNP-1	55.5±2.0°	28.8±1.1°	40.0±3.6°	29.8±2.4°	21.8±1.0°	31.17	1.74	50.27
AlgMNP-5	38.9±0.4°	25.6±1.9°	56.2±3.9°	27.4±1.0°	24.3±1.2°	40.16	0.34	54.14
AlgMNP-10	43.0±2.5°	23.3±2.1°	37.2±2.7°	23.4±1.5°	17.9±2.3°	38.05	1.22	48.56
AlgMNP-20	40.1±2.3°	16.6±3.0°	37.3±3.1°	25.5±0.6°	33.5±1.5°	39.56	0.24	61.87

Table S4. Optical density (545 nm) of the supernatant fluid (ODS) obtained by centrifugation of a suspension of erythrocytes pre-incubated with AlgMNP composite films with an area of (S_1) 0.25 cm^2 and (S_2) 0.56 cm^2.

Sample	S_1=0.25 см^2		S_2=0.56 см^2	
	ODS, a.u.	DEH, %	ODS, a.u.	DEH, %
AlgF	0.163±0.022	0.90±0.37	0.181±0.039	0.59±0.59
AlgMNP-1	0.111±0.022	0.26±0.15	0.193±0.026	0.38±0.30
AlgMNP-5	0.166±0.023	1.30±0.61	0.182±0.028	0.00±0.00
AlgMNP-10	0.167±0.016	0.96±0.24	0.244±0.047	1.08±0.62
AlgMNP-20	0.122±0.018	0.29±0.17	0.181±0.023	0.16±0.12

Note: DEH – degree of erythrocyte hemolysis; K ”-” – instead of the film, 10 mM phosphate-buffered saline pH 7.4, ODS for S_1 = 0.130±0.013 a.u., for S_2 = 0.223±0.028 a.u.; K “+” – instead of the film, distilled water, ODS = 4±0; n=5.

3. References

[1] C.J. Van Oss, M.K. Chaudhury, R.J. Good, Interfacial Lifshitz-van der Waals and polar interactions in macroscopic systems, Chem. Rev. 88 (1988) 927–941. doi:10.1021/cr00088a006.

[2] Y.. Ko, B.. Ratner, A.. Hoffman, Characterization of hydrophilic—hydrophobic polymeric surfaces by contact angle measurements, J. Colloid Interface Sci. 82 (1981) 25–37. doi:10.1016/0021-9797(81)90120-X.

[3] C.. Van Oss, L. Ju, M.. Chaudhury, R.. Good, Estimation of the polar parameters of the surface tension of liquids by contact angle measurements on gels, J. Colloid Interface Sci. 128 (1989) 313–319. doi:10.1016/0021-9797(89)90345-7.

[4] T. Çaykara, S. Demirci, M.S. Eroğlu, O. Güven, Poly(ethylene oxide) and its blends with sodium alginate, Polymer (Guildf). 46 (2005) 10750–10757. doi:10.1016/j.polymer.2005.09.041.

[5] T. Mosmann, Rapid colorimetric assay for cellular growth and survival: Application to proliferation and cytotoxicity assays, J. Immunol. Methods. 65 (1983) 55–63. doi:10.1016/0022-1759(83)90303-4.

[6] G.L. Gulliani, B.H. Hyun, M.B. Litten, Blood Recalcification Time: A Simple and Reliable Test to Monitor Heparin Therapy, Am. J. Clin. Pathol. 65 (1976) 390–396. doi:10.1093/ajcp/65.3.390.

[7] R.K. Stuart, A. Michel, Monitoring heparin therapy with the activated partial thromboplastin time., Can. Med. Assoc. J. 104 (1971) 385–8. http://www.ncbi.nlm.nih.gov/pubmed/5557913.

[8] B.C. Dash, G. Réthoré, M. Monaghan, K. Fitzgerald, W. Gallagher, A. Pandit, The influence of size and charge of chitosan/polyglutamic acid hollow spheres on cellular internalization, viability and blood compatibility, Biomaterials. 31 (2010) 8188–8197. doi:10.1016/j.biomaterials.2010.07.067.
